# Neural Dynamics as Sampling: A Model for Stochastic Computation in Recurrent Networks of Spiking Neurons

**DOI:** 10.1371/journal.pcbi.1002211

**Published:** 2011-11-03

**Authors:** Lars Buesing, Johannes Bill, Bernhard Nessler, Wolfgang Maass

**Affiliations:** Institute for Theoretical Computer Science, Graz University of Technology, Graz, Austria; Indiana University, United States of America

## Abstract

The organization of computations in networks of spiking neurons in the brain is still largely unknown, in particular in view of the inherently stochastic features of their firing activity and the experimentally observed trial-to-trial variability of neural systems in the brain. In principle there exists a powerful computational framework for stochastic computations, probabilistic inference by sampling, which can explain a large number of macroscopic experimental data in neuroscience and cognitive science. But it has turned out to be surprisingly difficult to create a link between these abstract models for stochastic computations and more detailed models of the dynamics of networks of spiking neurons. Here we create such a link and show that under some conditions the stochastic firing activity of networks of spiking neurons can be interpreted as probabilistic inference via Markov chain Monte Carlo (MCMC) sampling. Since common methods for MCMC sampling in distributed systems, such as Gibbs sampling, are inconsistent with the dynamics of spiking neurons, we introduce a different approach based on non-reversible Markov chains that is able to reflect inherent temporal processes of spiking neuronal activity through a suitable choice of random variables. We propose a neural network model and show by a rigorous theoretical analysis that its neural activity implements MCMC sampling of a given distribution, both for the case of discrete and continuous time. This provides a step towards closing the gap between abstract functional models of cortical computation and more detailed models of networks of spiking neurons.

## Author Summary

It is well-known that neurons communicate with short electric pulses, called action potentials or spikes. But how can spiking networks implement complex computations? Attempts to relate spiking network activity to results of deterministic computation steps, like the output bits of a processor in a digital computer, are conflicting with findings from cognitive science and neuroscience, the latter indicating the neural spike output in identical experiments changes from trial to trial, i.e., neurons are “unreliable”. Therefore, it has been recently proposed that neural activity should rather be regarded as *samples* from an underlying *probability distribution* over many variables which, e.g., represent a model of the external world incorporating prior knowledge, memories as well as sensory input. This hypothesis assumes that networks of stochastically spiking neurons are able to emulate powerful algorithms for reasoning in the face of uncertainty, i.e., to carry out probabilistic inference. In this work we propose a detailed neural network model that indeed fulfills these computational requirements and we relate the spiking dynamics of the network to concrete probabilistic computations. Our model suggests that neural systems are suitable to carry out probabilistic inference by using stochastic, rather than deterministic, computing elements.

## Introduction

Attempts to understand the organization of computations in the brain from the perspective of traditional, mostly deterministic, models of computation, such as attractor neural networks or Turing machines, have run into problems: Experimental data suggests that neurons, synapses, and neural systems are inherently *stochastic* [1], especially in vivo, and therefore seem less suitable for implementing deterministic computations. This holds for ion channels of neurons [2], synaptic release [3], neural response to stimuli (trial-to-trial variability) [4], [5], and perception [6]. In fact, several experimental studies arrive at the conclusion that external stimuli only modulate the highly stochastic spontaneous firing activity of cortical networks of neurons [7], [8]. Furthermore, traditional models for neural computation have been challenged by the fact that typical sensory data from the environment is often noisy and ambiguous, hence requiring neural systems to take *uncertainty* about external inputs into account. Therefore many researchers have suggested that information processing in the brain carries out probabilistic, rather than logical, inference for making decisions and choosing actions [9]–[22]. Probabilistic inference has emerged in the 1960’s [23], as a principled mathematical framework for reasoning in the face of uncertainty with regard to observations, knowledge, and causal relationships, which is characteristic for real-world inference tasks. This framework has become tremendously successful in real-world applications of artificial intelligence and machine learning. A typical computation that needs to be carried out for probabilistic inference on a high-dimensional joint distribution 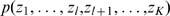 is the evaluation of the conditional distribution 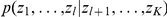 (or marginals thereof) over some variables of interest, say 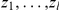, given variables 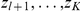. In the following, we will call the set of variables 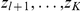, which we condition on, the *observed* variables and denote it by 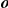.

Numerous studies in different areas of neuroscience and cognitive science have suggested that probabilistic inference could explain a variety of computational processes taking place in neural systems (see [10], [11]). In models of perception the observed variables 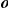 are interpreted as the sensory input to the central nervous system (or its early representation by the firing response of neurons, e.g., in the LGN in the case of vision), and the variables 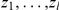 model the interpretation of the sensory input, e.g., the texture and position of objects in the case of vision, which might be encoded in the response of neurons in various higher cortical areas [15]. Furthermore, in models for motor control the observed variables 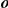 often consist not only of sensory and proprioceptive inputs to the brain, but also of specific goals and constraints for a planned movement [24]–[26], whereas inference is carried out over the variables 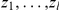 representing a motor plan or motor commands to muscles. Recent publications show that human reasoning and learning can also be cast into the form of probabilistic inference problems [27]–[29]. In these models learning of concepts, ranging from concrete to more abstract ones, is interpreted as inference in lower and successively higher levels of hierarchical probabilistic models, giving a consistent description of inductive learning within and across domains of knowledge.

In spite of this active research on the functional level of neural processing, it turned out to be surprisingly hard to relate the computational machinery required for probabilistic inference to experimental data on neurons, synapses, and neural systems. There are mainly two different approaches for implementing the computational machinery for probabilistic inference in “neural hardware”. The first class of approaches builds on deterministic methods for evaluating exactly or approximately the desired conditional and/or marginal distributions, whereas the second class relies on sampling from the probability distributions in question. Multiple models in the class of deterministic approaches implement algorithms from machine learning called message passing or belief propagation [30]–[33]. By clever reordering of sum and product operators occurring in the evaluation of the desired probabilities, the total number of computation steps are drastically reduced. The results of subcomputations are propagated as "messages" or "beliefs" that are sent to other parts of the computational network. Other deterministic approaches for representing distributions and performing inference are probabilistic population code (PPC) models [34]. Although deterministic approaches provide a theoretically sound hypothesis about how complex computations can possibly be embedded in neural networks and explain aspects of experimental data, it seems difficult (though not impossible) to conciliate them with other aspects of experimental evidence, such as stochasticity of spiking neurons, spontaneous firing, trial-to-trial variability, and perceptual multistability.

Therefore other researchers (e.g., [16]–[18], [35]) have proposed to model computations in neural systems as probabilistic inference based on a different class of algorithms, which requires stochastic, rather than deterministic, computational units. This approach, commonly referred to as sampling, focuses on drawing *samples*, i.e., concrete values for the random variables that are distributed according to the desired probability distribution. Sampling can naturally capture the effect of apparent stochasticity in neural responses and seems to be furthermore consistent with multiple experimental effects reported in cognitive science literature [17], [18]. On the conceptual side, it has proved to be difficult to implement learning in message passing and PPC network models. In contrast, following the lines of [36], the sampling approach might be well suited to incorporate learning.

Previous network models that implement sampling in neural networks are mostly based on a special sampling algorithm called Gibbs (or general Metropolis-Hastings) sampling [9], [17], [18], [37]. The dynamics that arise from this approach, the so-called Glauber dynamics, however are only superficially similar to spiking neural dynamics observed in experiments, rendering these models rather abstract. Building on and extending previous models, we propose here a family of network models, that can be shown to exactly sample from any arbitrary member of a well-defined class of probability distributions via their inherent network dynamics. These dynamics incorporate refractory effects and finite durations of postsynaptic potentials (PSPs), and are therefore more biologically realistic than existing approaches. Formally speaking, our model implements Markov chain Monte Carlo (MCMC) sampling in a spiking neural network. In contrast to prior approaches however, our model incorporates irreversible dynamics (i.e., no detailed balance) allowing for finite time PSPs and refractory mechanisms. Furthermore, we also present a continuous time version of our network model. The resulting stochastic dynamical system can be shown to sample from the correct distribution. In general, continuous time models arguably provide a higher amount of biological realism compared to discrete time models.

The paper is structured in the following way. First we provide a brief introduction to MCMC sampling. We then define the neural network model whose neural activity samples from a given class of probability distributions. The model will be first presented in discrete time together with some illustrative simulations. An extension of the model to networks of more detailed spiking neuron models which feature a relative refractory mechanism is presented. Furthermore, it is shown how the neural network model can also be formulated in continuous time. Finally, as a concrete simulation example we present a simple network model for perceptual multistability.

## Results

### Recapitulation of MCMC sampling

In machine learning, sampling is often considered the “gold standard” of inference methods, since, assuming that we can sample from the distribution in question, and assuming enough computational resources, any inference task can be carried out with arbitrary precision (in contrast to some deterministic approximate inference methods such as variational inference). However sampling from an arbitrary distribution can be a difficult problem in itself, as, e.g., many distributions can only be evaluated modulo a global constant (the partition function). In order to circumvent these problems, elaborate MCMC sampling techniques have been developed in machine learning and statistics [38]. MCMC algorithms are based on the following idea: instead of producing an ad-hoc sample, a process that is heuristically comparable to a global search over the whole state space of the random variables, MCMC methods produce a new sample via a “local search” around a point in the state space that is already (approximately) a sample from the distribution.

More formally, a Markov chain 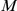 (in discrete time) is defined by a set 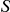 of states (we consider for discrete time only the case where 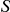 has a finite size, denoted by 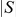) together with a transition operator 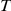. The operator 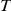 is a conditional probability distribution 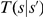 over the next state 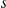 given a preceding state 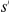. The Markov chain 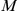 is started in some initial state 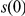, and moves through a trajectory of states 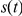 via iterated application of the stochastic transition operator 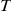. More precisely, if 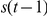 is the state at time 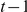, then the next state 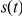 is drawn from the conditional probability distribution 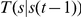. An important theorem from probability theory (see, e.g., p. 232 in [39]) states that if 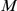 is irreducible (i.e., any state in 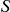 can be reached from any other state in 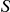 in finitely many steps with probability 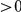) and aperiodic (i.e., its state transitions cannot be trapped in deterministic cycles), then the probability 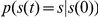 converges for 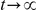 to a probability 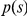 that does not depend on the initial state 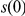. This state distribution 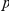 is called the invariant distribution of 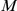. The irreducibility of 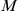 implies that it is the only distribution over the states 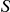 that is invariant under its transition operator 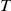, i.e.
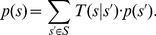
(1)


Thus, in order to carry out probabilistic inference for a given distribution 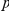, it suffices to construct an irreducible and aperiodic Markov chain 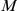 that leaves 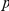 invariant, i.e., satisfies equation (1). Then one can answer numerous probabilistic inference questions regarding 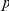 without any numerical computations of probabilities. Rather, one plugs in the observed values for some of the random variables (RVs) and simply collects samples from the conditional distribution over the other RVs of interest when the Markov chain approaches its invariant distribution.

A convenient and popular method for the construction of an operator 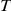 for a given distribution 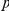 is looking for operators 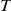 that satisfy the following detailed balance condition,
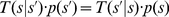
(2)for all 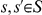. A Markov chain that satisfies (2) is said to be reversible. In particular, the Gibbs and Metropolis-Hastings algorithms employ reversible Markov chains. A very useful property of (2) is that it implies the invariance property (1), and this is in fact the standard method for proving (1). However, as our approach makes use of irreversible Markov chains as explained below, we will have to prove (1) directly.

### Neural sampling

Let 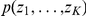 be some arbitrary joint distribution over 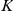 binary variables 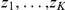 that only takes on values 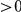. We will show that under a certain computability assumption on 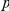 a network 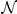 consisting of 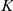 spiking neurons 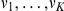 can sample from 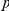 using its inherent stochastic dynamics. More precisely, we show that the stochastic firing activity of 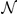 can be viewed as a non-reversible Markov chain that samples from the given probability distribution 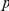. If a subset 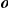 of the variables are observed, modelled as the corresponding neurons being “clamped” to the observed values, the remaining network samples from the conditional distribution of the remaining variables given the observables. Hence, this approach offers a quite natural implementation of probabilistic inference. It is similar to sampling approaches which have already been applied extensively, e.g., in Boltzmann machines, however our model is more biologically realistic as it incorporates aspects of the inherent temporal dynamics and spike-based communication of a network of spiking neurons. We call this approach *neural sampling* in the remainder of the paper.

In order to enable a network 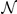 of spiking neurons to sample from a distribution 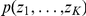 of binary variables 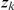, one needs to specify how an assignment 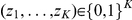 of values to these binary variables can be represented by the spiking activity of the network 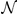 and vice versa. A spike, or action potential, of a biological neuron 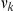 has a short duration of roughly 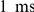. But the effect of such spike, both on the neuron 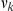 itself (in the form of refractory processes) and on the membrane potential of other neurons (in the form of postsynaptic potentials) lasts substantially longer, on the order of 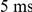 to 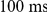. In order to capture this temporally extended effect of each spike, we fix some parameter 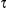 that models the average duration of these temporally extended processes caused by a spike. We say that a binary vector 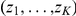 is represented by the firing activity of the network 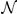 at time 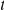 for 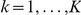 iff:
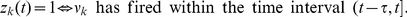
(3)


In other words, any spike of neuron 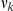 sets the value of the associated binary variable 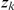 to 1 for a duration of length 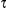.

An obvious consequence of this definition is that the binary vector 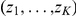 that is defined by the activity of 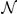 at time 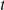 does not fully capture the internal state of this stochastic system. Rather, one needs to take into account additional non-binary variables 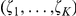, where the value of 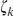 at time 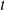 specifies *when* within the time interval 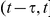 the neuron 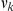 has fired (if it has fired within this time interval, thereby causing 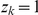 at time 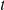). The neural sampling process has the Markov property only with regard to these more informative auxiliary variables 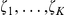. Therefore our analysis of neural sampling will focus on the temporal evolution of these auxiliary variables. We adopt the convention that each spike of neuron 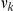 sets the value of 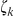 to its maximal value 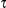, from which it linearly decays back to 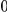 during the subsequent time interval of length 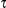.

For the construction of the sampling network 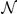, we assume that the membrane potential 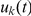 of neuron 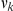 at time 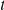 equals the log-odds of the corresponding variable 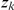 to be active, and refer to this property as *neural computability condition*:
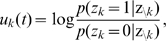
(4)where we write 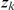 for 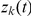 and 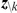 for the current values 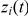 of all other variables 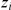 with 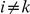. Under the assumption we make in equation (4), i.e., that the neural membrane potential reflects the log-odds of the corresponding variable 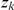, it is required that each single neuron in the network can actually compute the right-hand side of equation (4), i.e., that it fulfills the neural computability condition.

A concrete class of probability distributions, that we will use as an example in the remainder, are Boltzmann distributions:
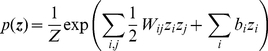
(5)with arbitrary real valued parameters 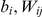 which satisfy 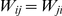 and 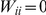 (the constant 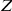 ensures the normalization of 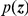). For the Boltzmann distribution, condition (4) is satisfied by neurons 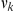 with the standard membrane potential
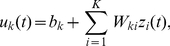
(6)where 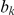 is the bias of neuron 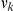 (which regulates its excitability), 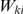 is the strength of the synaptic connection from neuron 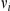 to 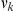, and 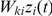 approximates the time course of the postsynaptic potential in neuron 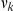 caused by a firing of neuron 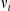 with a constant signal of duration 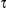 (i.e., a square pulse). As we will describe below, spikes of neuron 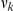 are evoked stochastically depending on the current membrane potential 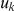 and the auxiliary variable 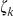.

The neural computability condition (4) links classes of probability distributions to neuron and synapse models in a network of spiking neurons. As shown above, Boltzmann distributions satisfy the condition if one considers point neuron models which compute a linear weighted sum of the presynaptic inputs. The class of distributions can be extended to include more complex distributions using a method proposed in [40] which is based on the following idea. Neuron 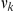 representing the variable 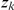 is not directly influenced by the activities 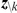 of the presynaptic neurons, but via intermediate nonlinear preprocessing elements. This preprocessing might be implemented by dendrites or other (inter-) neurons and is assumed to compute nonlinear combinations of the presynaptic activities 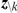 (similar to a kernel). This allows the membrane potential 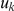, and therefore the log-odds ratio on the right-hand side of (4), to represent a more complex function of the activities 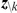, giving rise to more complex joint distributions 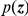. The concrete implementation of non-trivial directed and undirected graphical models with the help of preprocessing elements in the neural sampling framework is subject of current research. For the examples given in this study, we focus on the standard form of the membrane potential (6) of point neurons. As shown below, these spiking network models can emulate any Boltzmann machine (BM) [36].

A substantial amount of preceding studies has demonstrated that BMs are very powerful, and that the application of suitable learning algorithms for setting the weights 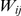 makes it possible to learn and represent complex sensory processing tasks by such distributions [37], [41]. In applications in statistics and machine learning using such Boltzmann distributions, sampling is typically implemented by Gibbs sampling or more general *reversible* MCMC methods. However, it is difficult to model some neural processes, such as an absolute refractory period or a postsynaptic potential (PSP) of fixed duration, using a reversible Markov chain, but they are more conveniently modelled using an irreversible one. As we wish to keep the computational power of BMs and at the same time to augment the sampling procedure with aspects of neural dynamics (such as PSPs with fixed durations, refractory mechanisms) to increase biological realism, we focus in the following on irreversible MCMC methods (keeping in mind that this might not be the only possible way to achieve these goals).

### Neural sampling in discrete time

Here we describe neural dynamics in discrete time with an absolute refractory period 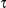. We interpret one step of the Markov chain as a time step 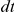 in biological real time. The dynamics of the variable 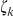, that describes the time course of the effect of a spike of neuron 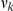, are defined in the following way. 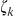 is set to the value 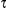 when neuron 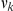 fires, and decays by 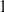 at each subsequent discrete time step. The parameter 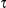 is chosen to be some integer, so that 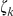 decays back to 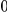 in exactly 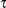 time steps. The neuron can only spike (with a probability that is a function of its current membrane potential 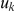) if its variable 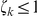. If however, 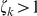, the neuron is considered refractory and it cannot spike, but its 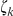 is reduced by 1 per time step. To show that these simple dynamics do indeed sample from the given distribution 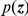, we proceed in the following way. We define a joint distribution 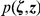 which has the desired marginal distribution 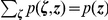. Further we formalize the dynamics informally described above as a transition operator 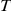 operating on the state vector 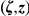. Finally, in the Methods section, we show that 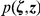 is the unique invariant distribution of this operator 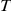, i.e., that the dynamics described by 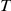 produce samples 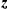 from the desired distribution 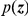. We refer to sampling through networks with this stochastic spiking mechanism as *neural sampling with absolute refractory period* due to the persistent refractory process.

Given the distribution 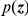 that we want to sample from, we define the following joint distribution 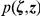 over the neural variables:
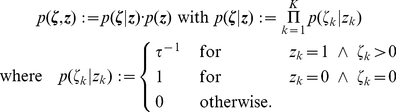
(7)


This definition of 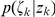 simply expresses that if 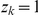, then the auxiliary variable 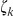 can assume any value in 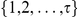 with equal probability. On the other hand 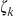 necessarily assumes the value 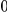 if 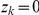 (i.e., when the neuron is in its resting state).

**Figure 1:**
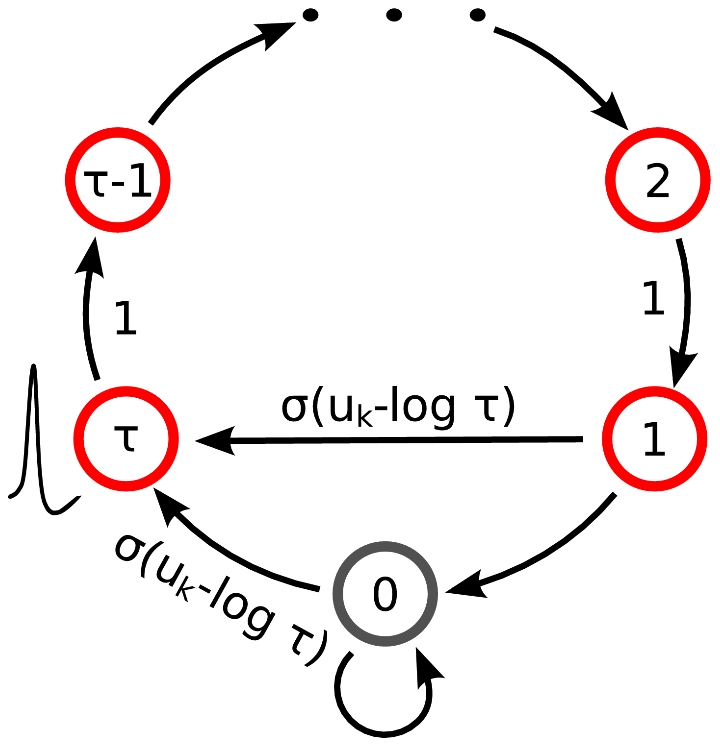
Neuron model with absolute refractory mechanism. The figure shows a schematic of the transition operator 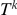 for the internal state variable 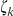 of a spiking neuron 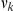 with an absolute refractory period. The neuron can fire in the resting state 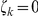 and in the last refractory state 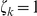.

The state transition operator 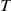 can be defined in a transparent manner as a composition of 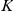 transition operators, 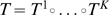, where 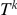 only updates the variables 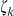 and 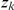 of neuron 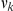, i.e., the neurons are updated sequentially in the same order (this severe restriction will become obsolete in the case of continuous time discussed below). We define the composition as 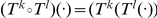, i.e., 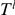 is applied prior to 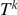. The new values of 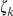 and 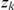 only depend on the previous value 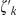 and on the current membrane potential 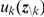. The interesting dynamics take place in the variable 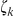. They are illustrated in Figure 1 where the arrows represent transition probabilities greater than 0.

If the neuron 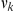 is not refractory, i.e., 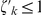, it can spike (i.e., a transition from 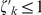 to 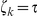) with probability
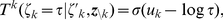
(8)where 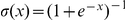 is the standard sigmoidal activation function and the 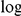 denotes the natural logarithm. The term 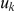 is the current membrane potential, which depends on the current values of the variables 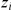 for 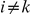. The term 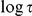 in (8) reflects the granularity of a chosen discrete time scale. If it is very fine (say one step equals one microsecond), then 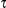 is large, and the firing probability at each specific discrete time step is therefore reduced. If the neuron in a state with 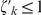 does not spike, 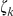 relaxes into the resting state 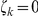 corresponding to a non-refractory neuron.

If the neuron is in a refractory state, i.e., 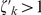, its new variable 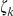 assumes deterministically the next lower value 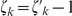, reflecting the inherent temporal process:
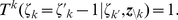
(9)


After the transition of the auxiliary variable 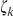, the binary variable 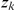 is deterministically set to a consistent state, i.e., 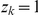 if 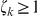 and 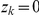 if 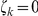.

It can be shown that each of these stochastic state transition operators 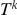 leaves the given distribution 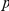 invariant, i.e., satisfies equation (1). This implies that any composition or mixture of these operators 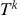 also leaves 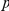 invariant, see, e.g., [38]. In particular, the composition 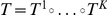 of these operators 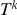 leaves 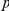 invariant, which has a quite natural interpretation as firing dynamics of the spiking neural network 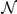: At each discrete time step the variables 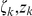 are updated for all neurons 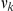, where the update of 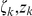 takes preceding updates for 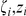 with 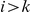 into account. Alternatively, one could also choose at each discrete time step a different order for updates according to [38]. The assumption of a well-regulated updating policy will be overcome in the continuous-time limit, i.e., in case where the neural dynamics are described as a Markov jump process. In the methods section we prove the following central theorem:

#### Theorem 1


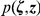
 *is the unique invariant distribution of operator* 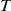, *i.e.,* 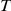 *is aperiodic and irreducible and satisfies*

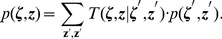
(10)


The proof of this Theorem is provided by Lemmata 1 – 3 in the Methods section. The statement that 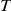 (which is composed of the operators 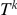) is irreducible and aperiodic ensures that 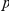 is the *unique* invariant distribution of the Markov chain defined by 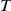, i.e., that irrespective of the initial network state the successive application of 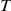 explores the whole state space in a non-periodic manner.

This theorem guarantees that after a sufficient “burn-in” time (more precisely in the limit of an infinite “burn-in” time), the dynamics of the network, which are given by the transition operator 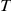, produce samples from the distribution 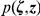. As by construction 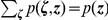, the Markov chain provides samples from the given distribution 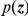. Furthermore, the network 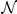 can carry out probabilistic inference for this distribution. For example, 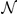 can be used to sample from the posterior distribution 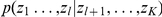 over 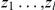 given 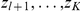. One just needs to clamp those neurons 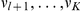 to the corresponding observed values. This could be implemented by injecting a strong positive (negative) current into the units with 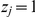 (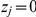). Then, as soon as the stochastic dynamics of 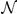 has converged to its invariant distribution, the averaged firing rate of neuron 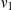 is proportional to the following desired marginal probability
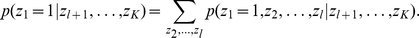



In a biological neural system this result of probabilistic inference could for example be read out by an integrator neuron that counts spikes from this neuron 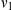 within a behaviorally relevant time window of a few hundred milliseconds, similarly as the experimentally reported integrator neurons in area LIP of monkey cortex [20], [21]. Another readout neuron that receives spike input from 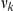 could at the same time estimate 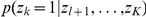 for another RV 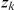. But valuable information for probabilistic inference is not only provided by firing rates or spike counts, but also by spike correlations of the neurons 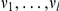 in 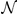. For example, the probability 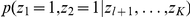 can be estimated by a readout neuron that responds to superpositions of EPSPs caused by near-coincident firing of neurons 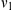 and 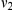 within a time interval of length 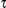. Thus, a large number of different probabilistic inferences can be carried out efficiently in parallel by readout neurons that receive spike input from different subsets of neurons in the network 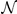.

#### Variation of the discrete time model with a relative refractory mechanism

For the previously described simple neuron model, the refractory process was assumed to last for 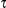 time steps, exactly as long as the postsynaptic potentials caused by each spike. In this section we relax this assumption by introducing a more complex and biologically more realistic neuron model, where the duration of the refractory process is decoupled from the duration 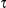 of a postsynaptic potential. Thus, this model can for example also fire bursts of spikes with an interspike interval 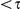. The introduction of this more complex neuron model comes at the price that one can no longer prove that a network of such neurons samples from the desired distribution 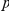. Nevertheless, if the sigmoidal activation function 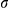 is replaced by a different activation function 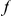, one can still prove that the sampling is “locally correct”, as specified in equation (12) below. Furthermore, our computer simulations suggest that also globally the error introduced by the more complex neuron model is not functionally significant, i.e. that statistical dependencies between the RVs 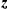 are still faithfully captured.

The neuron model with a relative refractory period is defined in the following way. Consider some arbitrary refractory function 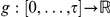 with 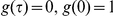, and 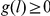 for 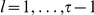. The idea is that 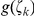 models the readiness of the neuron to fire in its state 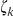. This readiness has value 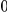 when the neuron has fired at the preceding time step (i.e., 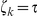), and assumes the resting state 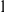 when 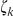 has dropped to 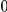. In between, the readiness may take on any non-negative value according to the function 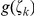. The function 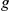 does not need to be monotonic, allowing for example that it increases to high values in between, yielding a preferred interspike interval of a oscillatory neuron. The firing probability of neuron 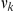 in state 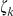 is given by 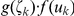, where 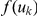 is an appropriate function of the membrane potential as described below. Thus this function 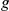 is closely related to the function 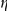 (called afterpotential) in the spike response model [5] as well as to the self-excitation kernel in Generalized Linear Models [42]. In general, different neurons in the network may have different refractory profiles, which can be modeled by a different refractory function for each neuron 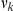. However for the sake of notational simplicity we assume a single refractory function in the following.

**Figure 2:**
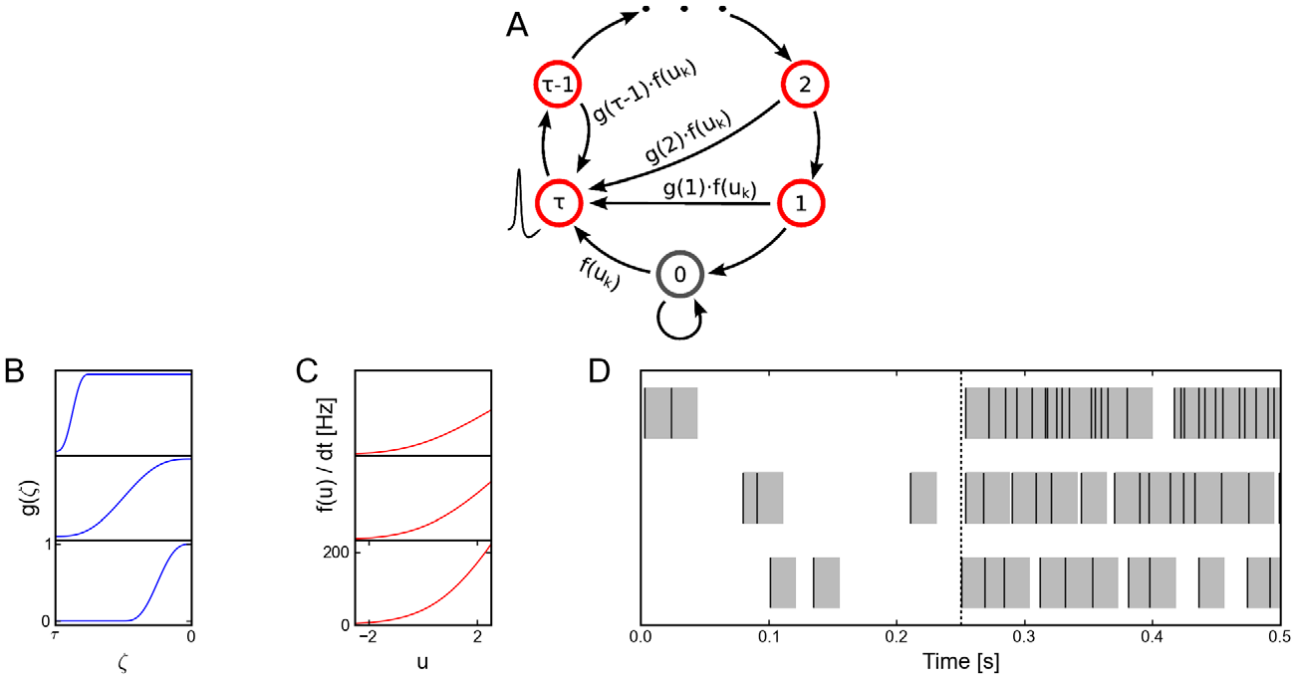
Neuron model with relative refractory mechanism. The figure shows the transition operator 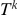, refractory functions 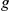 and activation functions 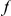 for the neuron model with relative refractory mechanism. (A) Transition probabilities of the internal variable 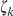 given by 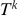. (B) Three examples of possible refractory functions 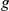. They assume value 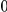 when the neuron cannot spike, and return to value 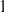 (full readiness to fire again) with different time courses. The value of 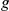 at intermediate time points regulates the current probability of firing of neuron 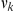 (see A). The x-axis is equivalent to the number of time steps since last spike (running from 0 to 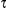 from left to right). (C) Associated activation functions 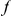 according to (11). (D) Spike trains produced by the resulting three different neuron models with (hypothetical) membrane potentials that jump at time 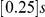 from a constant low value to a constant high value. Black horizontal bars indicate spikes, and the active states 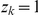 are indicated by gray shaded areas of duration 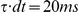 after each spike. It can be seen from this example that different refractory mechanisms give rise to different spiking dynamics.

In the presence of this refractory function 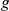 one needs to replace the sigmoidal activation function 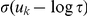 by a suitable function 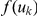 that satisfies the condition
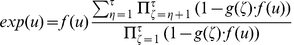
(11)for all real numbers 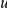. This equation can be derived (see Methods section Lemma 0) if one requires each neuron 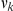 to represent the correct distribution 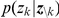 over 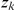 conditioned the variables 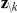. One can show that, for any 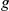 as above, there always exists a continuous, monotonic function 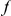 which satisfies this equation (see Lemma 0 in Methods). Unfortunately (11) cannot be solved analytically for 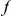 in general. Hence, for simulations we approximate the function 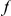 for a given 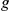 by numerically solving (11) on a grid and interpolating between the grid points with a constant function. Examples for several functions 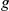 and the associated 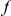 are shown in Figure 2B and Figure 2C respectively. Furthermore, spike trains emitted by single neurons with these refractory functions 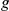 and the corresponding functions 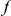 are shown in Figure 2D for the case of piecewise constant membrane potentials. This figure indicates, that functions 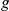 that define a shorter refractory effect lead to higher firing rates and more irregular firing. It is worth noticing that the standard activation function 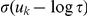 is the solution of equation (11) for the absolute refractory function, i.e., for 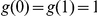 and 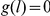 for 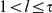.

The transition operator 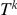 is defined for this model in a very similar way as before. However, for 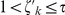, when the variable 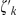 was deterministically reduced by 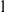 in the simpler model (yielding 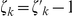), this reduction occurs now only with probability 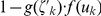. With probability 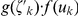 the operator 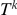 sets 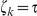, modeling the firing of another spike of neuron 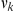 at this time point. The neural computability condition (4) remains unchanged, e.g., 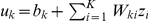 for a Boltzmann distribution. A schema of the stochastic dynamics of this local state transition operator 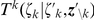 is shown in Figure 2A.

This transition operator 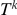 has the following properties. In Lemma 0 in Methods it is proven that the unique invariant distribution of 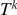, denoted as 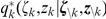, gives rise to the correct marginal distribution over 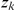, i.e.
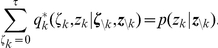



This means that a neuron whose dynamics is described by 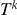 samples from the correct distribution 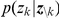 if it receives a static input from the other neurons in the network, i.e., as long as its membrane potential 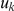 is constant. Hence the “local” computation performed by such neuron can be considered as correct. If however, several neurons in the network change their states in a short interval of time, the joint distribution over 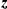 is in general not the desired one, i.e., 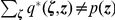, where 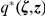 denotes the invariant distribution of 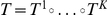. In the Methods section, we present simulation results that indicate that the error of the approximation to the desired Boltzmann distributions introduced by neural sampling with relative refractory mechanism is rather minute. It is shown that the neural sampling approximation error is orders of magnitudes below the one introduced by a fully factorized distribution (which amounts to assuming correct marginal distributions 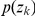 and independent neurons).

**Figure 3:**
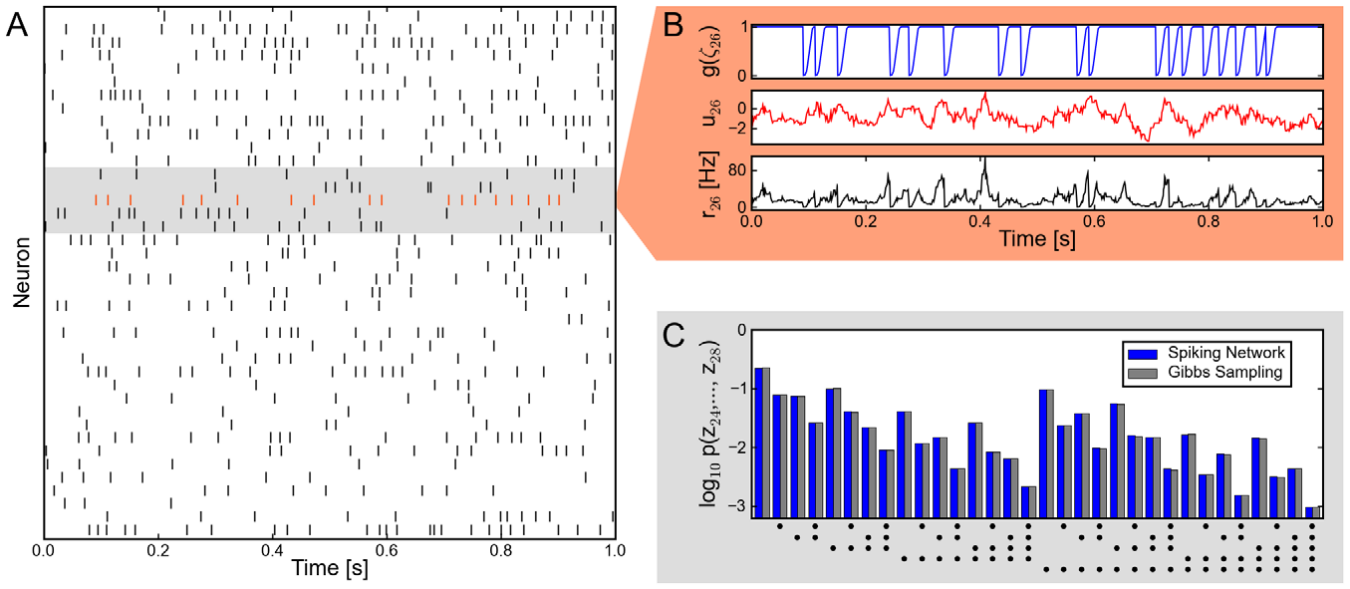
Sampling from a Boltzmann distribution by spiking neurons with relative refractory mechanism. (A) Spike raster of the network. (B) Traces of internal state variables of a neuron (# 26, indicated by orange spikes in A). The rich interaction of the network gives rise to rapidly changing membrane potentials and instantaneous firing rates. (C) Joint distribution of 5 neurons (gray shaded area in A) obtained by the spiking neural network and Gibbs sampling from the same distribution. Active states 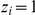 are indicated by a black dot, using one row for each neuron 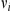, the columns list all 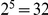 possible states 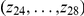 of these 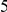 neurons. The tight match between both distributions suggests that the spiking network represents the target probability distribution 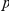 with high accuracy.

To illustrate the sampling process with the relative refractory mechanism, we examine a network of 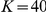 neurons. We aim to sample from a Boltzmann distribution (5) with parameters 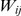, 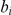 being randomly drawn from normal distributions. For the neuron model, we use the relative refractory mechanism shown in the mid row of Figure 2B. A detailed description of the simulation and the parameters used is given in the Methods section. A spike pattern of the resulting sampling network is shown in Figure 3A. The network features a sparse, irregular spike response with average firing rate of 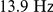. For one neuron 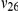, indicated with orange spikes, the internal dynamics are shown in Figure 3B. After each action potential the neuron’s refractory function 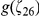 drops to zero and reduces the probability of spiking again in a short time interval. The influence of the remaining network 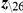 is transmitted to neuron 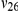 via PSPs of duration 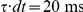 and sums up to the fluctuating membrane potential 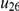. As reflected in the highly variable membrane potential even this small network exhibits rich interactions. To represent the correct distribution 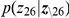 over 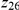 conditioned on 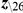, the neuron 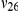 continuously adapts its instantaneous firing rate. To quantify the precision with which the spiking network draws samples from the target distribution (5), Figure 3C shows the joint distribution of 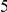 neurons. For comparison we accompany the distribution of sampled network states with the result obtained from the standard Gibbs sampling algorithm (considered as the ground truth). Since the number of possible states 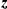 grows exponentially in the number of neurons, we restrict ourselves for visualization purposes to the distribution 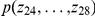 of the gray shaded units and marginalize over the remaining network. The probabilities are estimated from 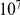 samples, i.e., from 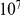 successive states 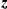 of the Markov chain. Stochastic deviations of the estimated probabilities due to the finite number of samples are quite small (typical errors 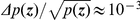) and are comparable to systematic deviations due to the only locally correct computation of neurons with relative refractory mechanism. In the Methods section, we present further simulation results showing that the proposed networks consisting of neurons with relative refractory mechanism approximate the desired target distributions faithfully over a large range of distribution parameters.

In order to illustrate that the proposed sampling networks feature biologically quite realistic spiking dynamics, we present in the Methods section several neural firing statistics (e.g., the inter-spike interval histogram) of the network model. In general, the statistics computed from the model match experimentally observed statistics well. The proposed network models are based on the assumption of rectangular-shaped, renewal PSPs. More precisely, we define renewal (or non-additive) PSPs in the following way. Renewal PSPs evoked by a single synapse do not add up but are merely prolonged in their duration (according to equation (6)); renewal PSPs elicited at different synapses nevertheless add up in the normal way. In Methods we investigate the impact of replacing the theoretically ideal rectangular-shaped, renewal PSPs with biologically more realistic alpha-shaped, additive PSPs. Simulation results suggest that the network model with alpha-shaped PSPs does not capture the target distribution as accurately as with the theoretically ideal PSP shapes, statistical dependencies between the RVs 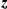 are however still approximated reasonably well.

### Neural sampling in continuous time

The neural sampling model proposed above was formulated in discrete time of step size 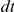, inspired by the discrete time nature of MCMC techniques in statistics and machine learning as well as to make simulations possible on digital computers. However, models in continuous time (e.g., ordinary differential equations) are arguably more natural and “realistic” descriptions of temporally varying biological processes. This gives rise to the question whether one can find a sensible limit of the discrete time model in the limit 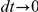, yielding a sampling network model in continuous time. Another motivation for considering continuous time models for neural sampling is the fact that many mathematical models for recurrent networks are formulated in continuous time [5], and a comparison to these existing models would be facilitated. Here we propose a stochastically spiking neural network model in continuous time, whose states still represent correct samples from the desired probability distribution 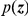 at any time 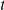. These types of models are usually referred to as Markov jump processes. It can be shown that discretizing this continuous time model yields the discrete time model defined earlier, which thus can be regarded as a version suitable for simulations on a digital computer.

We define the continuous time model in the following way. Let 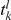, for 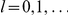, denote the firing times of neuron 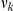. The refractory process of this neuron, in analogy to Figure 1 and equation (8)-(9) for the case of discrete time, is described by the following differential equation for the auxiliary variable 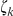, which may now assume any nonnegative real number 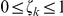:
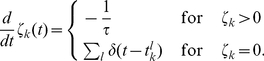
(12)


Here 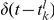 denotes Dirac’s Delta centered at the spike time 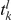. This differential equation describes the following simple dynamics. The auxiliary variable 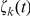 decays linearly with time constant 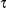 when the neuron is refractory, i.e., 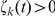. Once 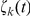 arrives at its resting state 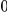 it remains there, corresponding to the neuron being ready to spike again (more precisely, in order to avoid point measures we set it to a random value in 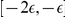, see Methods). In the resting state, the neuron has the probability density 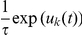 to fire at every time 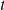. If it fires at 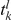, this results in setting 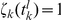, which is formalized in equation (12) by the sum of Dirac Delta’s 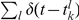. Here the current membrane potential 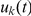 at time 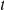 is defined as in the discrete time case, e.g., by 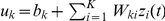 for the case of a Boltzmann distribution (5). The binary variable 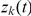 is defined to be 1 if 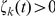 and 0 if the neuron is in the resting state 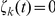. Biologically, the term 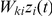 can again be interpreted as the value at time 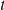 of a rectangular-shaped PSP (with a duration of 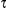) that neuron 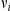 evokes in neuron 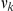. As the spikes are discrete events in continuous time, the probability of two or more neurons spiking at the same time is zero. This allows for updating all neurons in parallel using a differential equation.

In analogy to the discrete time case, the neural network in continuous time can be shown to sample from the desired distribution 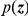, i.e., 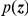 is an invariant distribution of the network dynamics defined above. However, to establish this fact, one has to rely on a different mathematical framework. The probability distribution 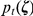 of the auxiliary variables 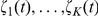 as a function of time 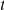, which describes the evolution of the network, obeys a partial differential equation, the so-called Differential-Chapman-Kolmogorov equation (see [43]):
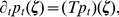
(13)where the operator 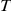, which captures the dynamics of the network, is implicitly defined by the differential equations (12) and the spiking probabilities. This operator 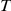 is the continuous time equivalent to the transition operator 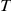 in the discrete time case. The operator 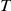 consists here of two components. The *drift term* captures the deterministic decay process of 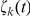, stemming from the term 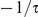 in equation (12). The *jump term* describes the non-continuous aspects of the path 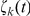 associated with “jumping” from 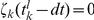 to 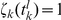 at the time 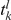 when the neuron fires.

In the Methods section we prove that the resulting time invariant distribution, i.e., the distribution that solves 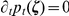, now denoted 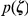 as it is not a function of time, gives rise to the desired marginal distribution 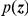 over 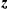:
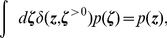
(14)where 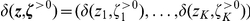 and 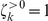 if 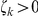 and 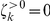 otherwise. 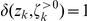 denotes Kronecker’s Delta with 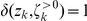 if 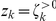 and 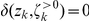 otherwise. Thus, the function 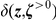 simply reflects the definition that 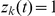 if 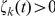 and 0 otherwise. For an explicit definition of 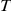, a proof of the above statement, and some additional comments see the Methods section.

The neural samplers in discrete and continuous time are closely related. The model in discrete time provides an increasingly more precise description of the inherent spike dynamics when the duration 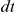 of the discrete time step is reduced, causing an increase of 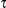 (such that 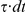 is constant) and therefore a reduced firing probability of each neuron at any discrete time step (see the term 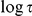 in equation (8)). In the limit of 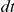 approaching 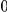, the probability that two or more neurons will fire at the same time approaches 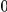, and the discrete time sampler becomes equal to the continuous time system defined above, which updates all units in parallel.

It is also possible to formulate a continuous time version of the neural sampler based on neuron models with relative refractory mechanisms. In the Methods section the resulting continuous time neuron model with a relative refractory mechanism is defined. Theoretical results similar to the discrete time case can be derived for this sampler (see Lemmata 9 and 10 in Methods): It is shown that each neuron “locally” performs the correct computation under the assumption of static input from the remaining neurons. However one can no longer prove in general that the global network samples from the target distribution 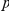.

### Demonstration of probabilistic inference with recurrent networks of spiking neurons in an application to perceptual multistability

In the following we present a network model for perceptual multistability based on the neural sampling framework introduced above. This simulation study is aimed at showing that the proposed network can indeed sample from a desired distribution and also perform inference, i.e., sample from the correct corresponding posterior distribution. It is not meant to be a highly realistic or exhaustive model of perceptual multistability nor of biologically plausible learning mechanisms. Such models would naturally require considerably more modelling work.

**Figure 4:**
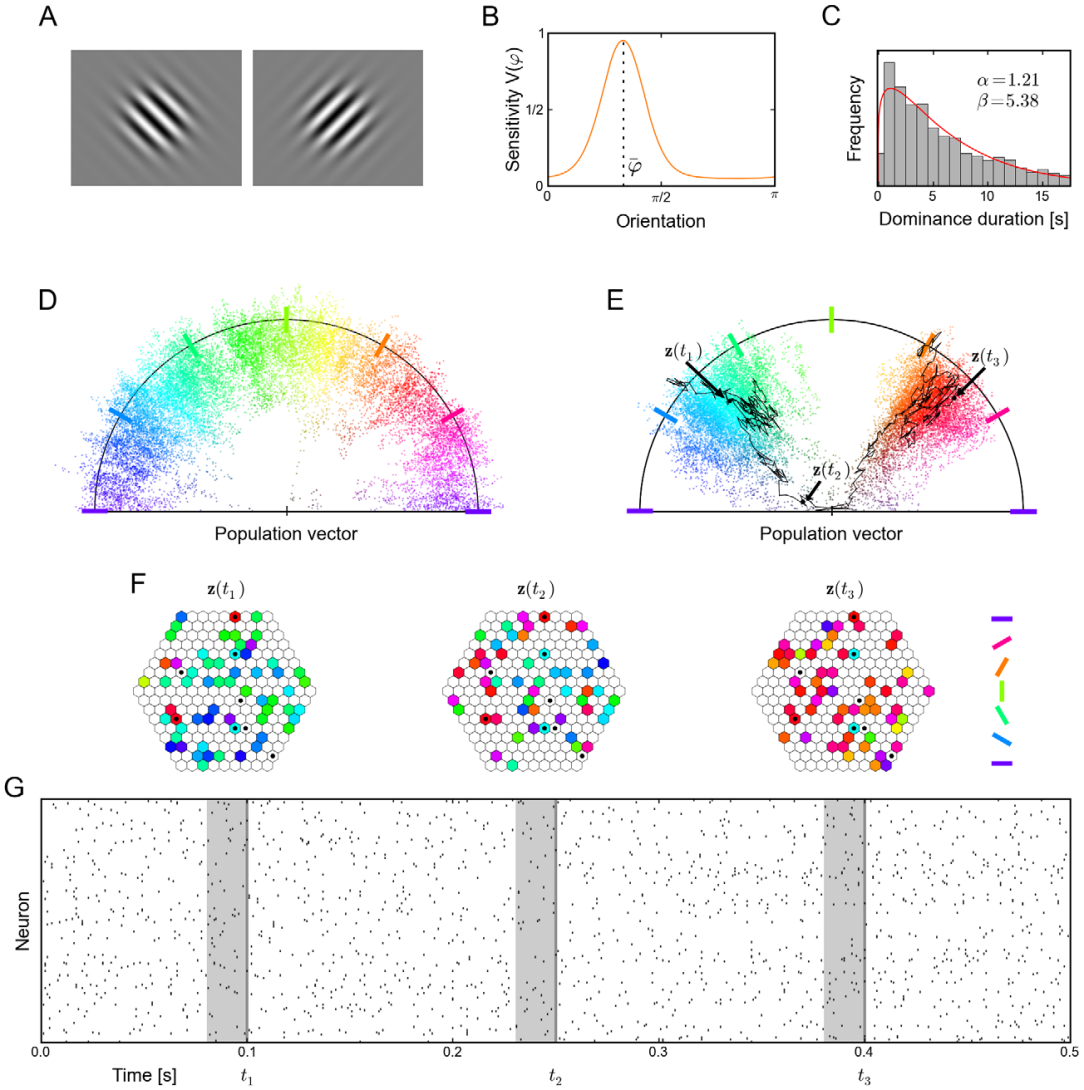
Modeling perceptual multistability as probabilistic inference with neural sampling. (A) Typical visual stimuli for the left and right eye in binocular rivalry experiments. (B) Tuning curve of a neuron with preferred orientation 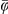. (C) Distribution of dominance durations in the trained network under ambiguous input. The red curve shows the Gamma distribution with maximum likelihood on the data. (D) 2-dimensional projection (via population vector) of the distribution 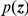 encoded in the spiking network showing that it strongly favors coherent global states of arbitrary orientation to incoherent ones (corresponding to population vectors of small magnitude). (E) 2-dimensional projection of the bimodal posterior distribution under an ambiguous input consisting of two different orientations reminiscent of the stimuli shown in A. The black trace shows the temporal evolution of the network state 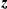 for 500 ms around a perceptual switch. (F) Network states at 3 time points 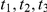 marked in E. Neurons that fired in the preceding 20 ms (see gray bar in G) are plotted in the color of their preferred orientation. Inactive neurons are shown in white. While states 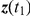 and 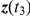 represent rather coherent orientations, 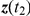 shows an incoherent state corresponding to a perceptual switch. Clamped neurons (which the posterior is condition on) are marked by a black dot. (G) Spike raster of the unclamped neurons during a 500 ms epoch marked by the black trace in E. Gray bars indicate the 20 ms time intervals that define the network states shown in F. Altogether this figure shows that a theoretically rigorous probabilistic inference process can be carried out by a network of spiking neurons with a spike raster that is similar to generic recorded data.

Perceptual multistability evoked by ambiguous sensory input, such as a 2D drawing (e.g., Necker cube) that allows for different consistent 3D interpretations, has become a frequently studied perceptual phenomenon. The most important finding is that the perceptual system of humans and nonhuman primates does not produce a superposition of different possible percepts of an ambiguous stimulus, but rather switches between different self-consistent global percepts in a spontaneous manner. Binocular rivalry, where different images are presented to the left and right eye, has become a standard experimental paradigm for studying this effect [44]–[47]. A typical pair of stimuli are the two images shown in Figure 4A. Here the percepts of humans and nonhuman primates switch (seemingly stochastically) between the two presented orientations. [16]–[18] propose that several aspects of experimental data on perceptual multistability can be explained if one assumes that percepts correspond to samples from the conditional distribution over interpretations (e.g., different 3D shapes) given the visual input (e.g., the 2D drawing). Furthermore, the experimentally observed fact that percepts tend to be stable on the time scale of seconds suggests that perception can be interpreted as probabilistic inference that is carried out by MCMC sampling which produces successively correlated samples. In [18] it is shown that this MCMC interpretation is also able to qualitatively reproduce the experimentally observed distribution of dominance durations, i.e., the distribution of time intervals between perceptual switches. However, in lack of an adequate model for sampling by a recurrent network of spiking neurons, theses studies could describe this approach only on a rather abstract level, and pointed out the open problem to relate this algorithmic approach to neural processes. We have demonstrated in a computer simulation that the previously described model for neural sampling could in principle fill this gap, providing a modelling framework that is on the one hand consistent with the dynamics of networks of spiking neurons, and which can on the other hand also be clearly understood from the perspective of probabilistic inference through MCMC sampling.

In the following we model some essential aspects of an experimental setup for binocular rivalry with grating stimuli (see Figure 4A) in a recurrent network of spiking neurons with the previously described relative refractory mechanism. We assigned to each of the 217 neurons in the network 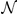 a tuning curve 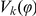, centered around its preferred orientation 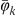 as shown in Figure 4B. The preferred orientations 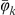 of the neurons were chosen to cover the entire interval 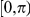 of possible orientations and were randomly assigned to the neurons. The neurons were arranged on a hexagonal grid as depicted in Figure 4F. Any two neurons with distance 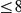 were synaptically connected (neighboring units had distance 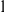). We assume that these neurons represent neurons in the visual system that have roughly the same or neighboring receptive field, and that each neuron receives visual input from either the left or the right eye. The network connections were chosen such that neurons that have similar (very different) preferred orientations are connected with positive (negative) weights (for details see Methods section).

We examined the resulting distribution 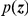 over the 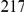 dimensional network states. To provide an intuitive visualization of these high dimensional network states 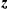, we resort to a 2-dimensional projection, the population vector of a state 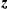 (see Methods for details of the applied population vector decoding scheme). Only the endpoints of the population vectors are drawn (as colored points) in Figure 4D,E. The orientation of the population vector is assumed to correspond to the dominant orientation of the percept, and its distance from the origin encodes the strength of this percept. We also, somewhat informally, call the strength of a percept its coherence and a network state which represents a coherent percept a coherent network state. A coherent network state hence results in a population vector of large magnitude. Each direction of a population vector is color coded in Figure 4D,E, using the color code for directions shown on the right hand side of Figure 4F. In Figure 4D the distribution 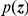 of the network is illustrated by sampling of the network for 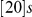, with samples 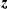 taken every millisecond. Each dot equals a sampled network state 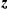. In a biological interpretation the spike response of the freely evolving network reflects spontaneous activity, since no observations, i.e., no external input, was added to the system. Figure 4D shows that the spontaneous activity of this simple network of spiking neurons moves preferably through coherent network states for all possible orientations due to the chosen recurrent network connections (being positive for neurons with similar preferred orientation and negative otherwise). This can directly be seen from the rare occurrence of population vectors with small magnitude (vectors close to the “center”) in Figure 4D.

To study percepts elicited by ambiguous stimuli, where inputs like in Figure 4A are shown simultaneously to the left and right eye during a binocular rivalry experiment, we provided ambiguous input to the network. Two cells with preferred orientation 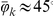 and two cells with 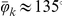 were clamped to 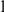. Additionally four neurons with 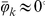 resp. 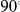 were muted by clamping to 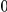. This ambiguous input is incompatible with a coherent percept, as it corresponds to two orthogonal orientations presented at the same time. The resulting distribution over the state of the 209 remaining neurons is shown for a time span of 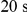 of simulated biological time (with samples taken every millisecond) in Figure 4E. One clearly sees that the network spends most of the time in network states that correspond to one of the two simultaneously presented input orientations (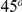 and 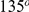), and virtually no time on orientations in between. This implements a sampling process from a bimodal conditional distribution. The black line marks a 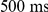 trace of network states 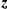 around a perceptual switch: The network remained in one mode of high probability – corresponding to one percept – for some period of time, and then quickly traversed the state space to another mode – corresponding to a different percept.

Three of the states 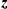 around this perceptual switch (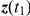, 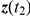 and 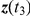 in Figure 4E) are explicitly shown in Figure 4F. Neurons 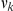 that fired during the preceding interval of 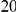 ms (marked in gray in Figure 4G) are drawn in the respective color of their preferred orientation. Inactive neurons are drawn in white, and clamped neurons are marked by a black dot (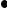).

Figure 4G shows the action potentials of the 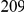 non-clamped neurons during the same 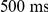 trace around the perceptual switch. One sees that the sampling process is expressed in this neural network model by a sparse, asynchronous and irregular spike response. It is worth mentioning that the average firing rate when sampling from the posterior distribution is only slightly higher than the average firing rate of spontaneous activity (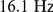 and 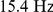 respectively), which is reminiscent of related experimental data [7]. Thus on the basis of the overall network activity it is indistinguishable whether the network carries out an inference task or freely samples from its prior distribution. It is furthermore notable, that a focus of the network activity on the two orientations that are given by the external input can be achieved in this model, in spite of the fact that only two of the 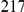 neurons were clamped for each of them. This numerical relationship is reminiscent of standard data on the weak input from LGN to V1 that is provided in the brain [48], [49], and raises the question whether the proposed neural sampling model could provide a possible mechanism (under the modelling assumptions made above) for cortical processing of such numerically weak external inputs.

The distribution of the resulting dominance durations, i.e., the time between perceptual switches, for the previously described setup with ambiguous input is shown for a continuous run of 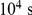 in Figure 4C (a similar method as in [18] was used to measure dominance durations, see Methods). This distribution can be approximated quite well by a Gamma distribution, which also provides a good fit to experimental data (see the discussion in [18]). We expect that also other features of the more abstract MCMC model for biological vision of [17], [18], such as contextual biases and traveling waves, will emerge in larger and more detailed implementations of the MCMC approach through the proposed neural sampling method in networks of spiking neurons.

## Discussion

We have presented a spiking neural network that samples from a given probability distribution via its inherent network dynamics. In particular the network is able to carry out probabilistic inference through sampling. The model, based on assumptions about the underlying probability distribution (formalized by the neural computability condition) as well as on certain assumptions regarding the underlying MCMC model, provides one possible neural implementation of the “inference-by-sampling paradigm” emerging in computational neuroscience.

**Figure 5:**
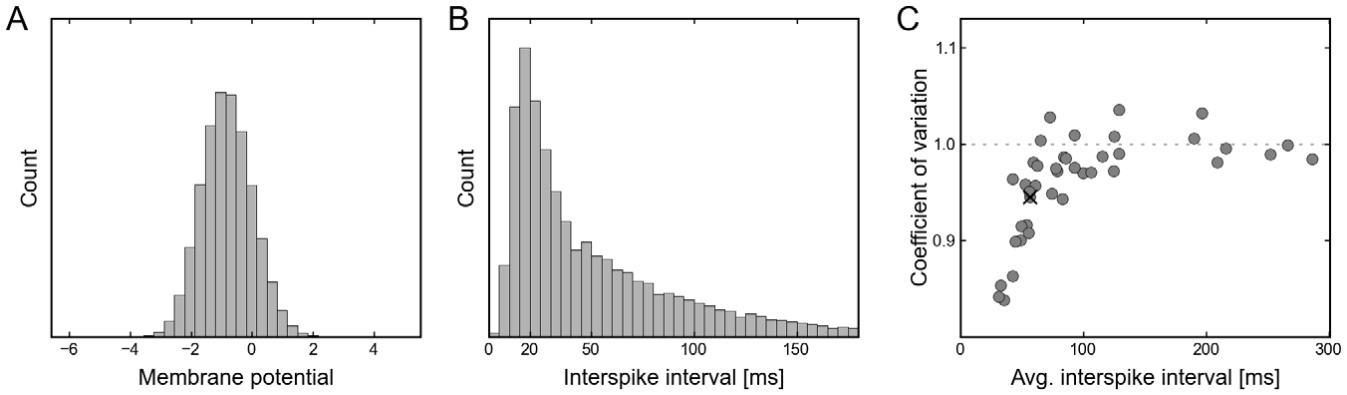
Firing statistics of neural sampling networks. (A) Shown is the membrane potential histogram of a typical neuron during sampling. The data is that of neuron 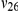 from the simulation shown in Figure 3 (the membrane potential and spike trace of 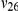 are highlighted in Figure 3). (B) The plot shows the ISI distribution of a typical neuron (again 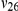 from Figure 3) during sampling. The distribution is roughly gamma-shaped, reminiscent of experimentally observed ISI distributions. (C) A scatter plot of the coefficient of variation (CV) versus the average interspike interval (ISI) of each neuron taken from the simulation shown in Figure 3. The value of neuron 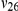 from Figure 3 is marked by a cross. The simulated data is in accordance with experimentally observed data.

During inference the observations (i.e., the variables which we wish to condition on) are modeled in this study by clamping the corresponding neurons by strong external input to the observed binary value. Units which receive no input or input with vanishing contrast (stimulus intensity) are treated as unobserved. Using this admittedly quite simplistic model of the input, we observed in simulations that our network model exhibits the following property: The onset of a sensory stimulus reduces the variability of the firing activity, which represents (after stimulus onset) a conditional distribution, rather than the prior distribution (see the difference between panels **D** and **E** of Figure 5. It is tempting to compare these results to the experimental finding of reduced firing rate variability after stimulus onset observed in several cortical areas [50]. We wish to point out however, that a consistent treatment of zero contrast stimuli requires more thorough modelling efforts (e.g., by explicitly adding a random variable for the stimulus intensity [35], [51]), which is not the focus of the presented work.

Virtually all high-level computational tasks that a brain has to solve can be formalized as optimization problems, that take into account a (possibly large) number of soft or hard constraints. In typical applications of probabilistic inference in science and engineering (see e.g. [52], [53]) such constraints are encoded in e.g., conditional probability tables or factors. In a biological setup they could possibly be encoded through the synaptic weights of a recurrent network of spiking neurons. The solution of such optimizations problems in a probabilistic framework via sampling, as implemented in our model, provides an alternative to deterministic solutions, as traditionally implemented in neural networks (see, e.g., [54] for the case of constraint satisfaction problems). Whereas an attractor neural network converges to *one* (possibly approximate) solution of the problem, a stochastic network may alternate between different approximate solutions and stay the longest at those approximate solutions that provide the best fit. This might be advantageous, as given more time a stochastic network can explore more of the state space and avoid shallow local minima. Responses to ambiguous sensory stimuli [44]–[47] might be interpreted as an optimization with soft constraints. The interpretation of human thinking as sampling process solving an inference task, recently proposed in cognitive science [28], [55], [56], further emphasizes that considering neural activity as an inferential process via sampling promises to be a fruitful approach.

Our approach builds on, and extends, previous work where recurrent networks of non-spiking stochastic neurons (commonly considered in artificial neural networks) were shown to be able to carry out probabilistic inference through Gibbs sampling [36]. In [57] a first extension of this approach to a network of recurrently connected spiking neurons had been presented. The dynamics of the recurrently connected spiking neurons are described as stepwise sampling from the posterior of a temporal Restricted Boltzmann Machine (tRBM) by introducing a clever interpretation of the temporal spike code as time varying parameters of a multivariate Gaussian distribution. Drawing one sample from the posterior of a RBM is, by construction, a trivial one-step task. In contrast to our model, the model of [57] does not produce multiple samples from a fixed posterior distribution, given the fixed input, but produces exactly one sample consisting of the temporal sequence of the hidden nodes, given a temporal input sequence. Similar temporal models, sometimes called Bayesian filtering, also underlie the important contributions of [58] and [32]. In [32] every single neuron is described as hidden Markov Model (HMM) with two states. Instead of drawing samples from the instantaneous posterior distribution using stochastic spikes, [32] presents a deterministic spike generation with the intention to convey the analog probability value rather than discrete samples. The approach presented here can be interpreted as a biologically more realistic version of Gibbs sampling for a specific class of probability distributions by taking into account a spike-based communication, finite duration PSPs and refractory mechanisms. Other implementations based on different distributions (e.g., directed graphical models) and different sampling methods (e.g., reversible MCMC methods) are of course conceivable and worth exploring.

In a computer experiment (see Figure 4, we used our proposed network to model aspects of biological vision as probabilistic inference along the lines of argumentation put forward in [16]–[18]. Our model was chosen to be quite simplistic, just to demonstrate that a number of experimental data on the dynamics of spontaneous activity [51], [59], [60] and binocular rivalry [44]–[47] can in principle be captured by this approach. The main point of the modelling study is to show that rather realistic neural dynamics can support computational functions rigorously formalized as inference via sampling.

We have also presented a model of spiking dynamics in continuous time that performs sampling from a given probability distribution. Although computer simulations of biological networks of neurons often actually use discrete time, it is desirable to also have a sound approach for understanding and describing the network sampling dynamics in continuous time, as the latter is arguable a natural framework for describing temporal processes in biology. Furthermore comparison to many existing continuous time neuron and network models of neurons is facilitated.

We have made various simplifying assumption regarding neural processes, e.g., simple symbolic postsynaptic potentials in the form of step-functions (reminiscent of plateau potentials caused by dendritic NMDA spikes [61]). More accurate models for neurons have to integrate a multitude of time constants that represent different temporal processes on the physical, molecular, and genetic level. Hence the open problem arises, to which extent this multitude of time constants and other complex dynamics can be integrated into theoretical models of neural sampling. We have gone one first step in this direction by showing that in computer simulations the two temporal processes that we have considered (refractory processes and postsynaptic potentials) can approximately be decoupled. Furthermore, we have presented simulation results suggesting that more realistic alpha-shaped, additive EPSPs are compatible with the functionality of the proposed network model.

Finally, we want to point out that the prospect of using networks of spiking neurons for probabilistic inference via sampling suggests new applications for energy-efficient spike-based and massively parallel electronic hardware that is currently under development [62], [63].

## Methods

We first provide details and proofs for the neural sampling models, followed by details for the computer simulations. Then we investigate typical firing statistics of individual neurons during neural sampling and examine the approximation quality of neural sampling with different neuron and synapse models.

### Mathematical details

#### Notation

To keep the derivations in a compact form, we introduce the following notations. We define the function 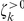 of 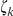 to be 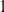 if 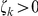 and 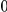 otherwise. Analogously we define 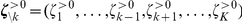. Let 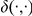 denote Kronecker’s Delta, i.e., 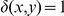 if 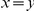 and 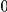 whereas 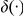 denotes Dirac’s Delta, i.e., 
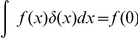
. Furthermore 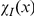 is the indicator function of the set 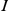, i.e., 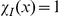 if 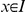 and 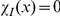 if 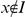.

#### Details to neural sampling with absolute refractory period in discrete time

The following Lemmata 1 – 3 provide a proof of Theorem 1. For completeness we begin this paragraph with a recapitulation of the definitions stated in Results. We then identify some central properties of the joint probability distribution 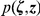 and proof that the proposed network samples from the desired invariant distribution.

For a given distribution 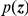 over the binary variables 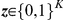 with 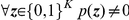, the joint distribution over 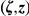 with 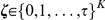 is defined in the following way (see equation 7):
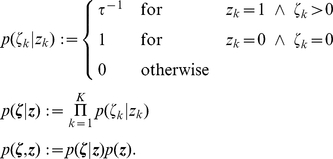



The assumption 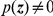 for all 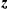 is required to show the irreducibility of the Markov chain, a prerequisite to ensure the uniqueness of the invariant distribution of the MCMC dynamics. Furthermore, for the given distribution 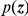 we define the functions 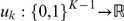 for 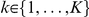 which map 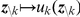:
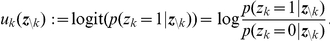



Instead of 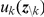 we simply write 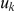 in the following.

*Lemma 1. The distribution* 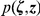 *has conditional distributions of the following form:*

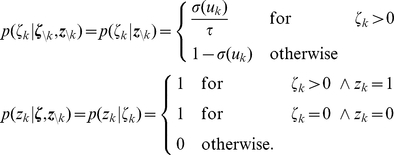



*These results can also be written more compactly in the following form:* 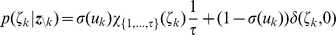 and 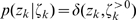.

*Proof.* Here we use the fact that the logistic function 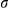 is the inverse of the logit function, i.e., 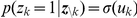.
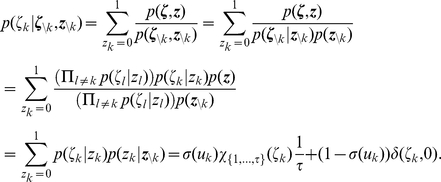



This also shows that 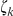 is independent from 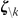 given 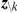, i.e., 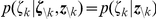. Now we show the second relation using Bayes’ rule:
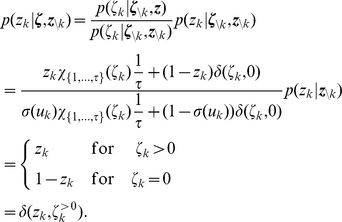



In order to facilitate the verification of the next two Lemmata, we first restate the definition of the operators 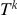 in a more concise way:
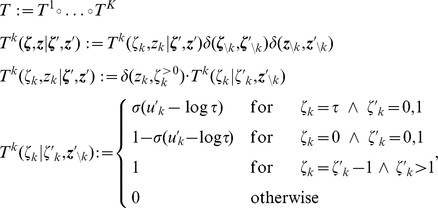
where 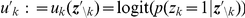.

*Lemma 2. For all* 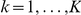 *the operator* 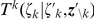 *leaves the conditional distribution* 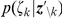 *invariant.*

*Proof.* For sake of simplicity, denote 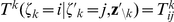 for 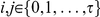 and 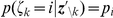. We have to show 
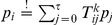
 for 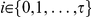.

First we show 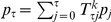 using 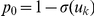 and 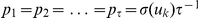 (which results from Lemma 1):
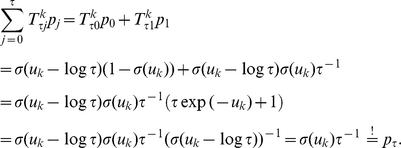



Here we used the definition of the logistic function 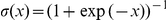 and 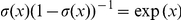.

Now we show 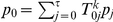:
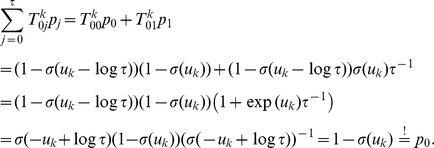



Here we used 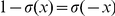.

It is trivial to show 
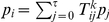
 for 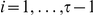 as 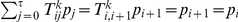. Here we used the facts that 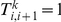 and 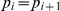 for 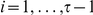 by definition.

*Lemma 3. For all* 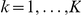 *the operator* 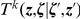 *leaves the distribution* 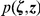 *invariant.*

*Proof.* We start from Lemma 2, which states that 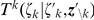 leaves the conditional distribution 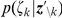 invariant:
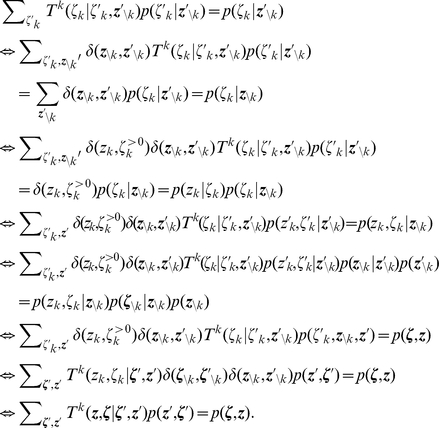



Here we used the relations 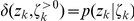 and 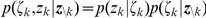 as well as 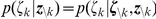 which directly follow from the definitions of 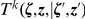 and 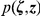.

Finally, we can verify that the composed operator 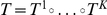 samples from the given distribution 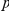.

*Theorem 1.* 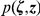 *is the unique invariant distribution of operator* 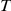 *.*

*Proof.* As all 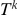 leave 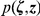 invariant, so does the concatenation 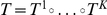. To ensure that 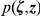 is the *unique* invariant distribution, we have to show that 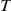 is irreducible and aperiodic. 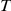 is aperiodic as the transition probabilities 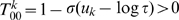 and 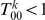 (this follows from the assumption 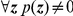 made above).

The operator 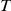 is also irreducible for the following reason. First we see that from any state 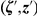 in at most 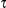 steps we can get to the zero-state 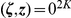 (and stay there) with non-zero probability, as 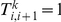 for 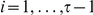 and 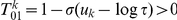. Furthermore, it can be seen that any state 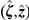 can be reached from the zero-state 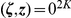 in at most 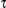 steps since 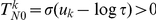 for any value of 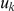. Hence every final state 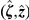 can be reached from every starting state 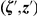 in at most 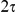 steps with non-vanishing probability.

#### Details to neural sampling with a relative refractory period in discrete time

We augment the neuron model with a relative refractory period described by a function 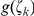. We first ensure existence of the corresponding function 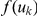. Based on these functions we then introduce the transition operator 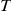 of the Markov chain. This operator is shown to entail correct “local” computations.

*Lemma 4. Let* 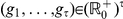 *be a tuple of non-negative real numbers, with* 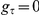 *and at least one element* 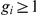 *. This defines the refractory function via* 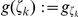 *. There exists a unique* 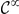 *function* 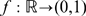 *with the following property* 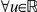 *:*

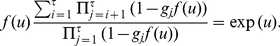
(15)


*Furthermore, the function* 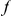 *has the property:*

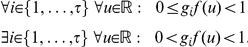



*Proof.* Let 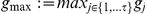; we know that 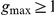. We define the function 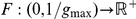:
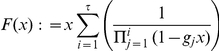



We can see that 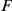 is a positive 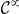 function on 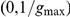. Furthermore, 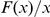 is defined as a sum of functions of the form 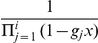. Each factor 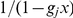 is positive and strictly monotonous. Therefore, 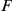 is strictly monotonous on 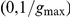 with the limits:
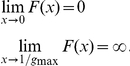



Hence the equation 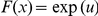 has a unique solution for 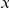 called 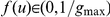 for all 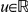. From applying the implicit function theorem to 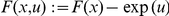 it follows that 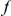 is 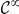.

From here on, with the letter 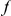 we will denote the function characterized by the above Lemma for the given tuple 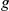 (which denotes the chosen refractory function).

*Definition 1. Define* 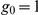 *. The transition operator* 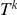 *is defined in the following way for all* 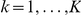:
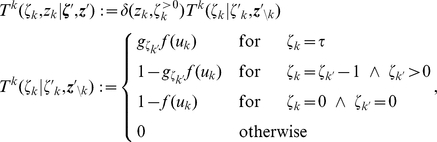

*with* 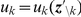.

*Lemma 5. For all* 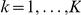 *the unique invariant distribution* 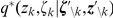 *of the operator* 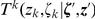 *fulfills* 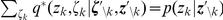 *. This means, for a constant configuration* 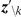 *, the operator* 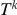 *produces samples* 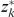 *from the correct conditional distribution* 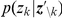 *.*

*Proof.* We define:
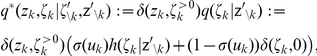
where the function 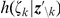 is defined as:
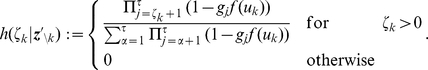



It is trivial to see that 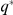 has the correct marginal distribution over 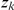:
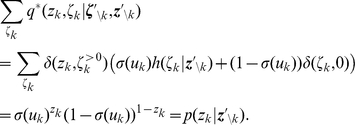



We now show that 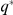 is the unique invariant distribution of 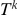. Because of the definition of 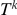, we only have to show that 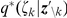 is the unique invariant distribution of 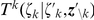. We denote 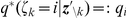 and 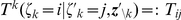, i.e., we have to show 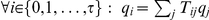.

It is trivial to show 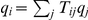 for 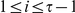, as there is only one non-vanishing element of transition operator, namely 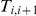:
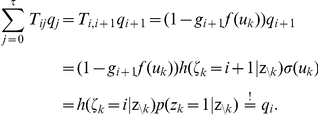



Here we used 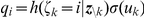 for 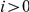 and the definition of 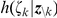.

Now we show 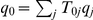 starting from equation (15) and additionally using the relations 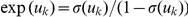 and 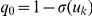 as well as the definition of 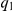. We define for the sake of simplicity 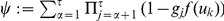:
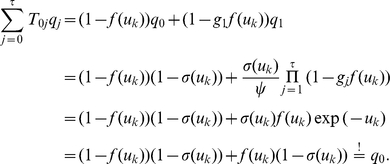



We finally show 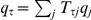, using the definition of 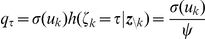:
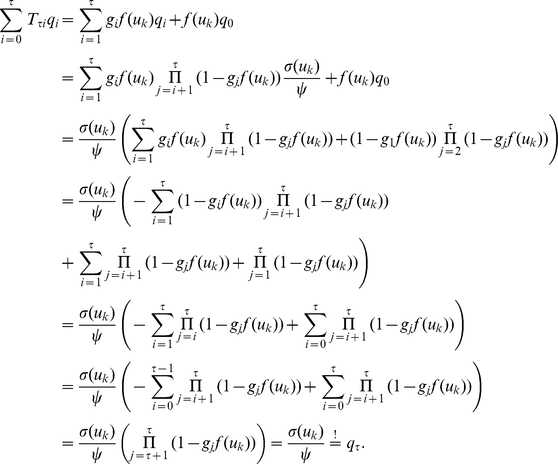



The argument that the transition operator 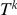 is aperiodic and irreducible is similar to the one presented in Lemma 1.

#### Details to neural sampling with an absolute refractory period in continuous time

In contrast to the discrete time model we define the state space of 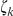 to be 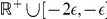 for 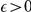, i.e., as the union of the positive real numbers and a small interval 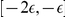. We will define the sampling operator in such a way that after neuron 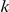 was refractory for exactly its refractory period 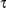, its refractory variable 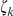 is uniformly placed in the small interval 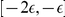, which represents now the resting state and replaces 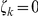. This avoids point measures (Dirac’s Delta) on the value 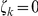. This system is still exactly equivalent to the system discussed in the main paper, as all spike-transition probabilities of 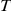 for 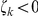 are constant. Hence, it does not matter which values 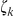 assumes with respect to the spike mechanism during its non-refractory period as long as 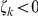.

*Definition 2. *For a given distribution 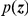 over the binary variables 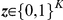 with 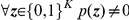, we define a joint distribution over 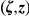 with 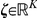 in the following way:
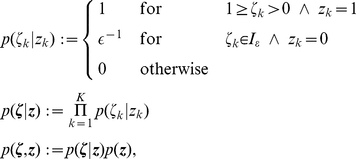

*where* 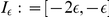 *is the refractory resting state interval. In accordance with this definition we can also write* 
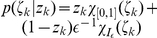
 *.*

*Lemma 6. The distribution* 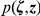 *has the following marginal distribution*:
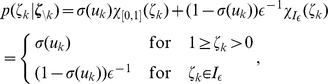
where 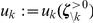.

*Definition 3. For* 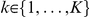 *and* 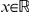 *the operator* 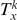 *is defined in the following way for a function* 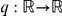:
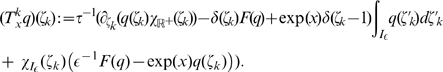

*where the functional* 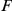 *is defined as the one-sided limit from above at 0:*

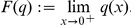



*The operator* 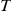 *is defined in the following way for a probability distribution* 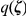 *on* 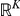 *:*

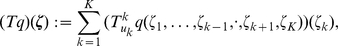

*where* 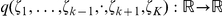 *denotes the function* 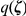 *of* 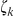 *where* 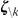 *is held constant and* 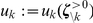 *.*

The transition operator 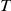 defines the following Fokker-Planck equation for a time-dependent distribution 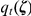:
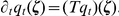



The jump and drift functions 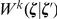 and 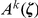 associated to the operator 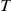 are given by:
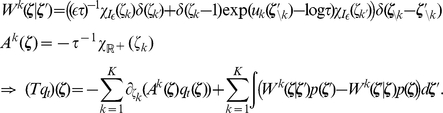



*Lemma 7. The operator* 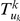 *leaves the conditional distribution* 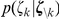 *invariant with* 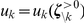 *, i.e.:*

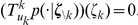



*Proof.* This is easy to proof using calculus and the relations 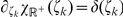 and 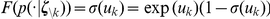.

*Lemma 8.* 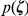 *is an invariant distribution of* 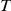 *, i.e., it is a solution to the invariant Fokker-Planck equation:*

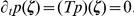



*Proof.* We observe that 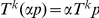 for a constant 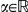 (which is not a function of 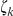). Hence:
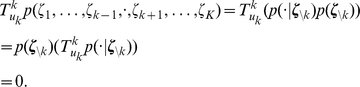



The Lemma follows then from the definition of 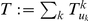.

#### Details to neural sampling with a relative refractory period in continuous time

As already assumed in the case of the absolute refractory sampler in continuous time, we define the state space of 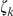 to be 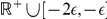 for 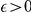.

*Lemma 9. Let* 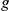 *be a continuous, non-negative function* 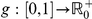 *with* 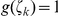 *for* 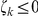 *. There exists a unique* 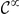 *function* 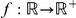 *with the following property* 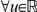 *:*

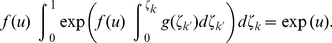
(16)


*Proof.* We define the function 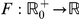 in the following way:
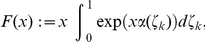
where 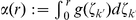. From 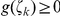 we can follow that 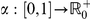 is non-negative. 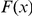 is differentiable with the derivative:
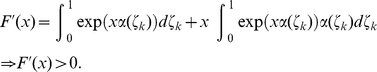



Hence 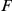 is strictly monotonously increasing. Furthermore, the following relations hold:
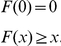



Therefore the equation:
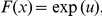
has exactly one solution 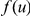 with 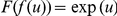 in 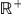. From applying the implicit function theorem to 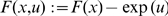 it follows that 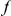 is 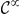.

*Definition 4. For all* 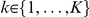 *and* 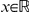 *the operator* 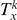 *is defined in the following way for a function* 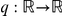:
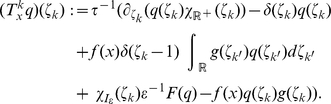



The transition operator 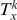 defines the following Fokker-Planck equation for a time-dependent distribution 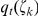:
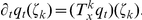



The jump and drift functions 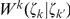 and 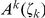 associated to the operator 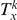 are given by:
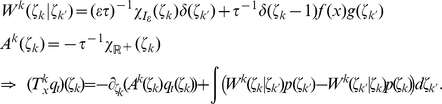



*Lemma 10. For all* 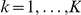 *the invariant distribution* 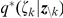 *of the operator* 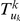 *fulfills* 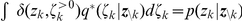.

*Proof.* We define the distribution 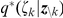 as:
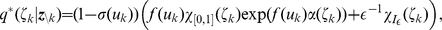
where 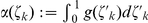. By applying the operator 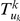 to 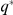 one can verify that 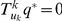 holds using the definition of 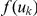 given in (16). Furthermore we can compute the ratio:
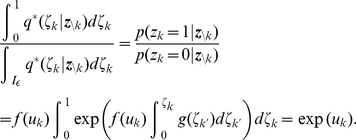



### Details to the computer simulations

The simulation results shown in Figure 2, Figure 3 and Figure 4 used the biologically more realistic neuron model with the relative refractory mechanism. During all experiments the first second of simulated time was discarded as burn-in time. The full list of parameters defining the experimental setup is given in Table 1. All occurring joint probability distributions are Boltzmann distributions of the form given in equation (5). Example Python [64] scripts for neural sampling from Boltzmann distributions are available on request and will be provided on our webpage. The example code comprises networks with both absolute and relative refractory mechanism. It requires standard Python packages only and is readily executable.

**Table 1:** List of parameters of the computer simulations.

Description	Variable	Value	Figure	Comment
*Simulation Time*				
Simulation step size	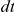	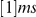	2–7	interpretation of an MCMC step
Burn-in time	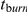	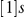	2–7	before recording spikes
Simulation time	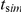	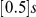	2	
		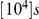	3,5–7	
		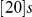	4	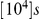 for Figure 3C
*Network*				
Number of neurons	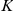	3	2	unconnected
		40	3,5,6	randomly connected
		217	4	
		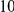	7	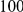 networks
Connection radius		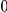	2	
		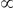	3,5–7	
		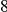	4	
Recurrent weights	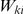	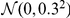	3,5–7	from Gaussian distribution
Falling edge	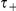	[20] *ms*	6,7	for realistic PSP shapes
Rising edge	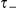	[3] *ms*	6,7	
Scaling factor	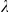	20/17	6,7	
*Neuron Model*				
Number recovery steps	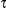	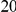	2–7	PSP duration 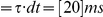
Refractory function	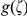	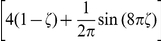	2 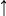	normalized to 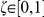 ,
		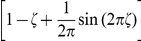	2–7	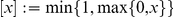
		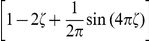	2 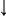 ,7	
Excitability	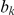	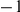 or 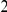	2	defines membrane potential 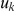
		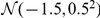	3,5–7	from Gaussian distribution
		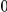	4	initial value
*Tuning Function, Training and Inference (* *Figure 4* *)*				
Peakedness	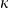	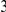	4	measured: 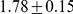
Base sensitivity	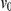	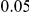	4	measured: 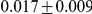
Sensitivity contrast	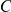	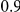	4	measured: 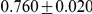
Training samples	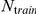	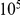	4	
Decorrelation steps		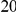	4	for contrastive divergence
Learning rate	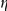	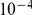	4	
Number of neurons clamped on/off		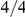	4	

#### Details to Figure 2: Neuron model with relative refractory mechanism

The three refractory functions 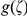 of panel (B) as well as all other simulation parameters are listed in Table 1. Panel (C) shows the corresponding functions 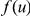, which result from numerically solving equation (11). The spike patterns in panel (D) show the response of the neurons when the membrane potential is low (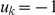 for 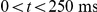) or high (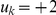 for 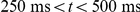). These membrane potentials encode 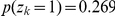 and 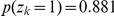, respectively according to (3) and (4). The binary state 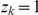 is indicated by gray shaded areas of duration 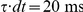 after each spike.

#### Details to Figure 3: Sampling from a Boltzmann distribution by spiking neurons with relative refractory mechanism

We examined the spike response of a network of 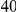 randomly connected neurons which sampled from a Boltzmann distribution. The excitabilities 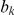 as well as the synaptic weights 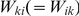 were drawn from Gaussian distributions (with diagonal elements 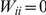). For the full list of parameters please refer to Table 1. One second of the arising spike pattern is shown in panel (A). The average firing rate of the network was 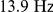. To highlight the internal dynamics of the neuron model, the values of the refractory function 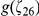, the membrane potential 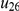 and the instantaneous firing rate 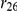 of neuron 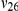 (indicated with red spikes) are shown in panel (B). Here, the instantaneous firing rate 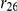 is defined for the discrete time Markov chain as
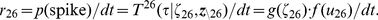
(17)


As stated before, the neuron model with relative refractory mechanism 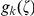 does not entail the correct overall invariant distribution 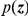. To estimate the impact of this approximation on the joint network dynamics, we compared the distribution 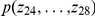 over five neurons (indicated by gray background in A) in the spiking network with the correct distribution obtained from Gibbs sampling. The probabilities were estimated from 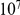 samples. A more quantitative analysis of the approximation quality of neural sampling with a relative refractory mechanism is provided below.

#### Details to Figure 4: Modeling perceptual multistability as probabilistic inference with neural sampling

We demonstrate probabilistic inference and learning in a network of orientation selective neurons. As a simple model we consider a network of 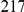 neurons on a hexagonal grid as shown in panel (F). Any two neurons with distance 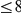 were synaptically connected (neighboring units had distance 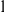). For the remaining parameters of the network and neuron model please refer to Table 1. Each neuron featured a 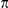-periodic tuning curve as depicted in panel (B):
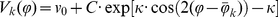
(18)with base sensitivity 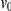, contrast 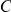, peakedness 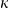 and preferred orientation 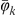. The preferred orientations 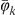 of the neurons were chosen to cover the entire interval 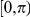 of possible orientations with equal spacing and were randomly assigned to the neurons.

For simplicity we did not incorporate the input dynamics in our probabilistic model, but rather trained the network directly like a fully visible Boltzmann machine. We used for this purpose a standard Boltzmann machine learning rule known as contrastive divergence [41], [65]. This learning rule requires posterior samples 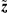, i.e., network states under the influence of the present input, and approximate prior samples 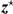, which reflect the probability distribution of the network in the absence of stimuli. The update rules for synaptic weights and neuronal excitabilities read:
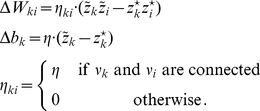
(19)


While more elaborate policies can speed up convergence, we simply used a global learning rate 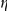 which was constant in time. The values of 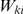 and 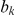 were initialized at 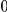. We generated binary training patterns in the following way:

1. A global orientation 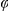 was drawn uniformly from 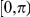,
2. each neuron was independently set to be active with probability 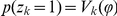,
3. the resulting network state 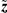 was taken as posterior sample.

To obtain an approximate prior sample 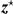 we let the network run for a short time freely starting from 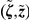. The variables 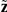 were also assumed to be observed with 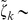 iid. uniformly in 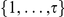 if 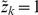 and 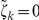 otherwise. After evolving freely for 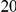 time steps, the resulting network state 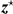 was taken as approximate prior sample and 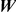 and 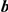 were updated according to (19). This process was repeated 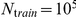 times. As a result, neurons with similar preferred orientations featured excitatory synaptic connections (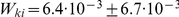  =  mean 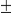 standard deviation of weight distribution), those with dissimilar orientations maintained inhibitory synapses (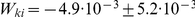). Here, preferred orientations 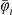 and 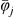 are defined as similar if 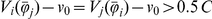, otherwise they are dissimilar. Neuronal biases converged to 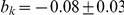.

We illustrate the learned prior distribution 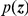 of the network through sampled states when the network evolved freely. As seen in panel (D), the population vector – a 2-dimensional projection of the high dimensional network state – typically reflected an arbitrary, yet coherent, orientation (for the definition of the population vector see below). Each dot represents a sampled network state 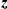.

To apply an ambiguous cue, we clamped 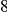 out of 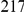 neurons: Two units with 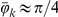 and two with 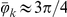 were set active, two units with 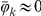 and two with 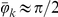 were set inactive. This led to a bimodal posterior distribution as shown in panel (E). The sampling network represented this distribution by encoding either global perception separately: The trace of network states 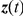 roamed in one mode for multiple steps before quickly crossing the state space towards the opposite percept.

We define the population vector 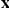 of a network state 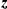 as a function of the preferred orientations of all active units:
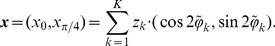
(20)


This definition of 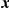 is not based on the preferred orientations 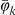 which are used for generating external input to the network from a given stimulus with orientation 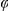. It is rather based on the preferred orientations 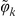 measured from the network response. We used population vector decoding based on the measured values 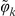, as they are conceptually closer to experimentally measurable preferred orientations, and this decoding hence does not require knowledge of the (unobservable) 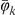. For every neuron 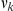 the preferred orientation 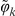 was measured in the following way. We estimated a tuning curve 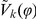 by a van-Mises fit (of the form (18)) to data from stimulation trials in which neuron 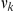 was not clamped, i.e., where 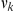 was only stimulated by recurrent input (feedforward input was modeled by clamping 8 out of 217 neurons as a function of stimulus orientation 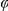 as before). Due to the structured recurrent weights, the experimentally measured tuning curves 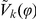 were found to be reasonably close to the tuning curves 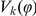 used for external stimulation. 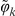 was set to the preferred orientation of 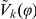 (localization parameter of the van-Mises fit). The measured values 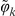 turned out to be consistent with the preferred orientations 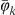 (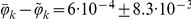 averaged over all 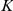 neurons). The mean and standard deviation of the remaining parameter values 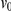, 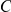 and 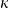 of the fitted tuning curves 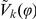 are listed in Table 1 next to the ones used for stimulation.

The population vector 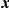 was defined in (20) with the argument 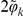 (instead of 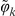) as orthogonal orientations should cancel each other and neighborhood relations should be respected. For example neurons with 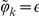 and 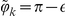 contribute similarly to the population vector for small 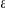. But counter to intuition the population vector of a state 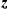 with dominant orientation 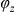 will point into direction 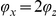. For visualization in panel (D) and (E) we therefore rescaled the population vector: If 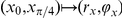 in polar coordinates, then the dot is located at 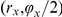 in accord with intuition. The black semicircles equal 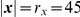.

The population vector 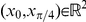 was also used for measuring the dominance durations shown in panel (C). To this 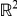 was divided into 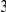 areas: (a) 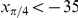, (b) 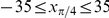, (c) 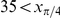. We detected a perceptual switch when the network state entered area (a) or (c) while the previous perception was (c) or (a), respectively.

In panel (F) neurons 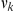 with 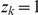 are plotted with their preferred orientation color code, inactive neurons are displayed in white. Cells marked by a dot (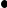) were part of the observed variables 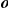. The three network states correspond to 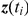 with 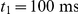, 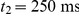 and 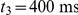 in the spike pattern in panel (G). The spike pattern shows the response of the freely evolving units around a perceptual switch during sampling from the posterior distribution. The corresponding trace of the population vector is drawn as black line in panel (E). The width of the light-gray shaded areas in the spike pattern equals the PSP duration 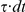, i.e., neurons that spiked in these intervals were active in the corresponding state in (F).

### Firing statistics of neural sampling networks

In previous sections it was shown that a spiking neural network can draw samples from a given joint distribution which is in a well-defined class of probability distributions (see the neural computability condition (4)). Here, we examine some statistics of individual neurons in a sampling network which are commonly used to analyze experimental data from recordings. The spike trains and membrane potential data are taken from the simulation presented in Figure 3.

Figure 5A,B exemplarily show the distribution of the membrane potential 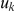 and the interspike interval (ISI) histogram of a single neuron, namely neuron 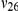 which was already considered in Figure 3B. The responses of other neurons yield qualitatively similar statistics. The bell-shaped distribution of the membrane potential is commonly observed in neurons embedded in an active network [66]. The ISI histogram reflects the reduced spiking probability immediately after an action potential due the refractory mechanism. Interspike intervals larger than the refractory time constant 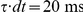 roughly follow an exponential distribution. Similar ISI distributions were observed during in-vivo recordings in awake, behaving monkeys [67].

Figure 5C shows a scatterplot of the coefficient of variation (CV) of the ISIs versus the average ISI for each neuron in the network. The neurons exhibited a variety of average firing rates between 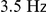 and 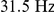. Most of the neurons responded in a highly irregular manner with a CV 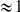. Neurons with high firing rates had a slightly lower CV due to the increased influence of the refractory mechanism The dashed line marks the CV of a Poisson process, i.e., a memoryless spiking behavior. The CV of neuron 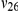 is marked by a cross. The structure of this plot resembles, e.g., data from recordings in behaving macaque monkeys [68] (but note the lower average firing rate).

### Approximation quality of neural sampling with different neuron and synapse models

The theory of the neuron model with absolute refractory mechanism guarantees sampling form the correct distribution. In contrast, the theory for the neuron model with a relative refractory mechanism only shows that the sampling process is “locally correct”, i.e., that it would yield correct conditional distributions 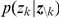 for each individual neuron if the state of the remaining network 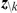 stayed constant. Therefore, the stationary distribution of the sampling process with relative refractory mechanism only provides an approximation to the target distribution. In the following we examine the approximation quality and robustness of sampling networks with different refractory mechanisms for target Boltzmann distributions with parameters randomly drawn from different distributions. Furthermore, we investigate the effect of additive PSP shapes with more realistic time courses.

We generated target Boltzmann distributions with randomly drawn weights 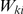 and biases (excitabilities) 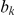 and computed the similarity between these reference distributions and the corresponding neural sampling approximations. The setup of these simulations is the same as for the simulation presented in Figure 3. As we aimed to compare the distribution 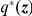 sampled by the network with the exact Boltzmann distribution 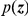, we reduced the number of neurons per network to 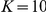. This resulted in a state space of 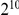 possible network states 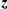 for which the normalization constant for the target Boltzmann distribution could be computed exactly. The weight matrix 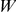 was constraint to be symmetric with vanishing diagonal. Off-diagonal elements were drawn from zero-mean normal distributions with three different standard deviations 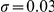, 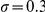 and 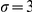, whereas the 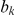 were sampled from the same distribution as in Figure 3. For every value of the hyperparameter 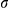 we generated 100 random distributions. For Boltzmann distributions with small weights (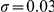), the RVs are nearly independent, whereas distributions with intermediate weights (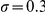) show substantial statistical dependencies between RVs. For very large weights (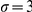), the probability mass of the distributions is concentrated on very few states (usually 90% on less than 10 out of the 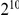 states). Hence, the range of the hyperparameter 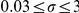 considered here covers a range a very different distributions.

The approximation quality of the sampled distribution was measured in terms of the Kullback-Leibler divergence between the target distribution 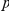 and the neural approximation 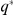


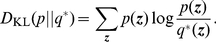
(21)


We estimated 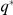 from 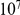 samples for each simulation trial using a Laplace estimator, i.e., we added a priori 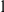 to the number of occurrences of each state 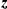.

Table 2 shows the means and the standard deviations of the Kullback-Leibler divergences between the target Boltzmann distributions and the estimated approximations stemming from neural sampling networks with three different neuron and synapse models: the exact model with absolute refractory mechanism and two models with different relative refractory mechanisms shown in the bottom and middle row in Figure 2B. Additionally, as a reference, we provide the (analytically calculated) Kullback-Leibler divergences for fully factorized distributions, i.e., 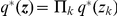 with correct marginals 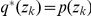 but independent variables 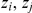 for 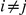.

**Table 2:** Approximation quality of networks with different refractory mechanisms.

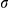	Absolute refractory	Rel. late recovery	Rel. moderate recovery	Prod. of marginals
0.03	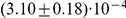	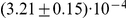	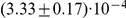	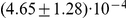
0.3	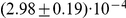	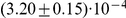	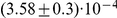	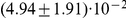
3.0	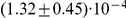	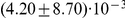	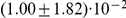	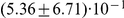

Mean and standard deviation of the Kullback-Leibler divergence 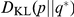 between reference Boltzmann distributions 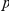 and neural sampling approximations 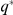 for three different neuron models (corresponding to columns) and three different values for the reference distribution hyperparameter 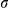 (corresponding to rows). The parameter 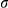 controls the standard deviation of the weights of the reference distributions 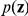. In case of very strong synaptic interactions (leading to sharply peaked distributions, 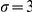) the approximation quality of the spiking network degrades, if the neurons feature a relative refractory mechanism. The data was computed from 100 randomly generated Boltzmann distributions and their neural approximations for each value of 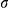.

**Figure 6:**
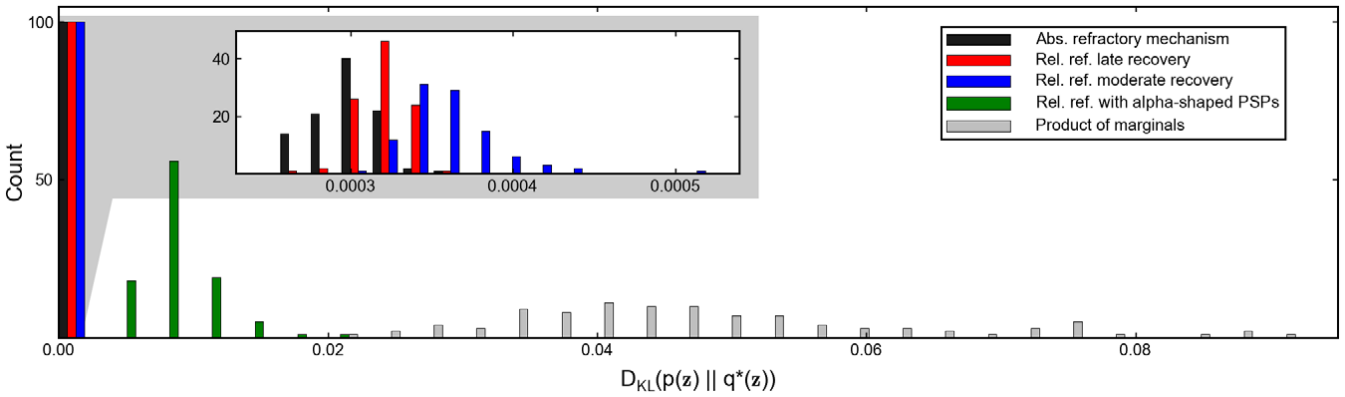
Comparison of neural sampling with different neuron and synapse models. The figure shows a histogram of the Kullback-Leibler divergence between 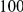 different Boltzmann distributions over K = 10 variables (with parameters randomly drawn, see setup of Figure 3) and approximations stemming from different neural sampling networks. Networks with absolute refractory mechanism provide the best approximation (as expected from theoretical guarantees). Networks consisting of neurons with relative refractory mechanisms, with only “locally” correct sampling, also provide a close fit to the true distribution (see inset) compared to a fully factorized approximation (assuming correct marginals and independent variables). Furthermore, it can be seen that sampling networks with more realistic, alpha-shaped, additive PSPs still fit the true distribution reasonably well.

The absolute refractory model provides the best results as we expected due to the theoretical guarantee to sample from the correct distribution (the non-zero Kullback-Leibler divergence is caused by the estimation from a finite number of samples). The models with relative refractory mechanism provide faithful approximations for all values of the hyperparameter 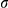 considered here. These relative refractory models are characterized by the theory to be “locally correct” and turn out to be much more accurate approximations than fully factorized distributions if substantial statistical dependencies between the RVs are present (i.e., 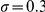, 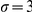). As expected, a late recovery of the refractory function 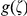 is beneficial for the approximation quality of the model as it is closer to an absolute refractory mechanism. Figure 6 shows the full histograms of the Kullback-Leibler divergences for the intermediate weights group (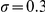). Systematic deviations due to the relative refractory mechanism are on the same order as the effect of estimating from finite samples (as can be seen, e.g., from a comparison with the absolute refractory model which has 0 systematic error). For completeness, we mention that the divergences of the fully factorized distributions of 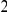 out of the 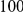 networks with 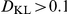 are not shown in the plot.

**Figure 7:**
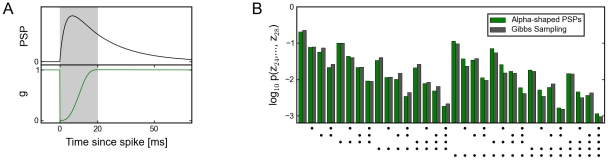
Sampling from a Boltzmann distribution with more realistic PSP shapes. (A) The upper panel shows the shape of a single PSP elicited at time 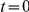. The lower panel shows the time course of the refractory function 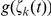 caused by a single spike of neuron 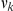 at 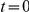. The grey-shaded area of length 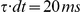 indicates the interval of neuron 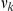 being active (i.e., 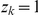) due to a single spike of neuron 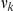 at time 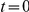. (B) Shown is the probability distribution of 5 out of 40 neurons. The plot is similar to Figure 3C, however it is generated with a sampling network that features alpha-shaped, additive PSPs. It can be seen that the network still produces a reasonable approximation to the true Boltzmann distribution (determined by Gibbs sampling).

The theorems presented in this article assumed renewed (i.e., non-additive), rectangular PSPs. In the following we examine the effect of additive PSPs with more realistic time courses. We define additive, alpha-shaped PSPs in the following way. The influence 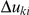 of each presynaptic neuron 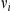 on the postsynaptic membrane potential 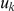 is modeled by convolving the input spikes with a kernel 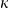:
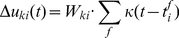
(22)where 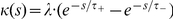 for 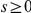 and 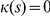 for 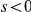, and 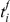 for 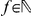 are the spike times of the presynaptic neuron 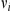. The time constant governing the rising edge of the PSPs was set to 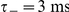. The time constant controlling the falling edge was chosen equal to the duration of rectangular PSPs, 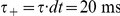. The scaling parameter 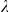 was set such that the time integral over a single PSP matches the time integral over the theoretically optimal rectangular PSP, i.e., 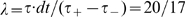. These parameters display a simple and reasonable choice for the purpose of this study (an optimization of 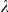, 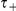 and 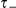 is likely to yield an improved approximation quality). Figure 7A shows the resulting shape of the non-rectangular PSP. Furthermore the time course of the function 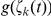 caused by a single spike of neuron 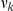 is shown in order to illustrate that the time constants of 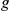 and of a PSP are closely related due to the assumption 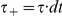 made above. Preliminary and non-exhaustive simulations seem to suggest that the choice 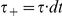 yields better approximation quality than setting 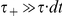 or 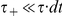; however it is very well possible that a mismatch between 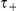 and 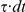 can be compensated for by adapting other parameters, e.g., the PSP magnitude or a specific choice of the refractory function 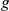. Figure 7B shows the results of an experiment, similar to the one presented in Figure 3C , with additive, alpha-shaped PSPs and relative refractory mechanism. While differences to Gibbs sampling results are visible, the spiking network still captures dependencies between the binary random variables quite well.

For a quantitative analysis of the approximation quality, we repeated the experiment of Figure 6 with additive, alpha-shaped PSPs (shown as green bars). The Kullback-Leibler divergence 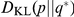 to the true distribution is clearly higher compared to the case of renewed, rectangular PSPs. Still networks with this more realistic synapse model account for dependencies between the random variables 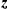 and yield a better approximation of 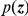 than fully factorized distributions.
